# Plant Reactome: a knowledgebase and resource for comparative pathway analysis

**DOI:** 10.1093/nar/gkz996

**Published:** 2019-11-04

**Authors:** Sushma Naithani, Parul Gupta, Justin Preece, Peter D’Eustachio, Justin L Elser, Priyanka Garg, Daemon A Dikeman, Jason Kiff, Justin Cook, Andrew Olson, Sharon Wei, Marcela K Tello-Ruiz, Antonio Fabregat Mundo, Alfonso Munoz-Pomer, Suhaib Mohammed, Tiejun Cheng, Evan Bolton, Irene Papatheodorou, Lincoln Stein, Doreen Ware, Pankaj Jaiswal

**Affiliations:** 1 Department of Botany & Plant Pathology, Oregon State University, Corvallis, OR, USA; 2 NYU School of Medicine, New York, NY, USA; 3 Ontario Institute for Cancer Research, Toronto, ON, Canada; 4 Cold Spring Harbor Laboratory, Cold Spring Harbor, NY, USA; 5 European Molecular Biology Laboratory - European Bioinformatics Institute, Hinxton, UK; 6 National Center for Biotechnology Information, National Library of Medicine, National Institutes of Health, Bethesda, MD 20894, USA; 7 USDA-ARS, RW Holley Center for Agriculture & Health, Ithaca, NY, USA

## Abstract

Plant Reactome (https://plantreactome.gramene.org) is an open-source, comparative plant pathway knowledgebase of the Gramene project. It uses *Oryza sativa* (rice) as a reference species for manual curation of pathways and extends pathway knowledge to another 82 plant species via gene-orthology projection using the Reactome data model and framework. It currently hosts 298 reference pathways, including metabolic and transport pathways, transcriptional networks, hormone signaling pathways, and plant developmental processes. In addition to browsing plant pathways, users can upload and analyze their omics data, such as the gene-expression data, and overlay curated or experimental gene-gene interaction data to extend pathway knowledge. The curation team actively engages researchers and students on gene and pathway curation by offering workshops and online tutorials. The Plant Reactome supports, implements and collaborates with the wider community to make data and tools related to genes, genomes, and pathways Findable, Accessible, Interoperable and Re-usable (FAIR).

## INTRODUCTION

**Figure 1.**
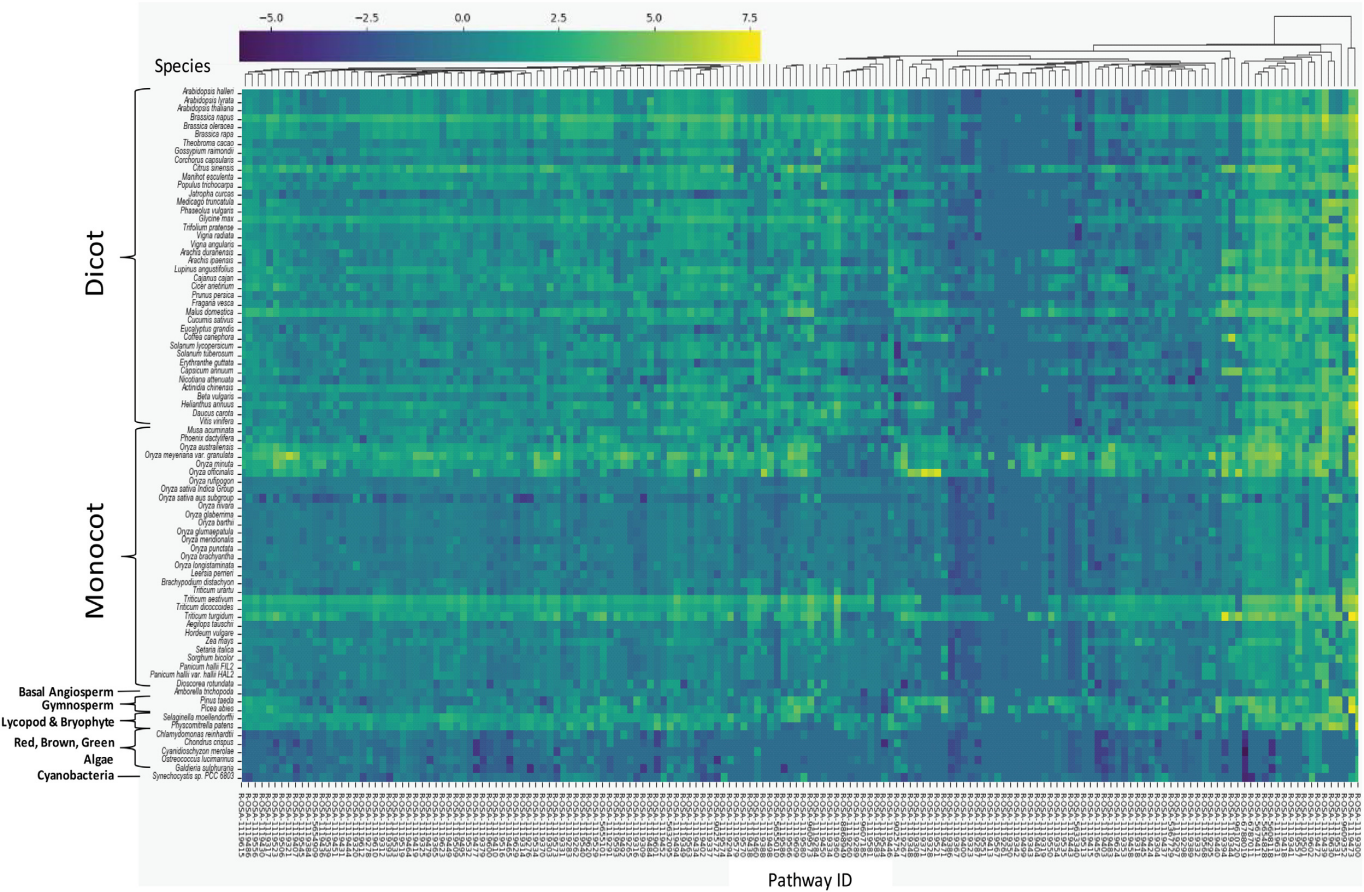
Gene orthology based pathway projections for the 82 plant species in the Plant Reactome. The pathways and associated reactions are clustered based on event hierarchy and species are ordered based on phylogeny. The shades of green to yellow are trending towards a higher number of ortholog count and the shades of blue trending towards lower counts of orthologs associated with individual pathways.

To meet growing demand for food, feedstock, and energy sustainably, plant breeders and agriculture scientists need new strategies to accelerate conventional plant breeding and synthetic biology-based crop improvement by integration and analysis of heterogeneous big data sets. The Gramene project (http://gramene.org) contributes to the establishment and stewardship of open data, specifically plant genomes and pathways, to assist plant scientists in accessing, analyzing and visualizing datasets to address important biological questions and formulate data-driven hypotheses (1). Plant Reactome (https://plantreactome.gramene.org) (2) is the Gramene's pathway knowledgebase that adopts the Reactome data model (3) to represent various types of reactions associated with plant pathways and biological processes in the context of their subcellular location within a plant cell. Plant Reactome pathways are manually curated for the reference species *Oryza sativa* (rice) to build the conceptual framework of a systems-level plant pathway network by associating small molecules, metabolites, gene products and macromolecular interactions with terminal pathways, which in turn are grouped into a higher-order event hierarchy (2). From the curated reference species, we provide pathway projections to other plant species using a gene-orthology based approach and allow researchers to traverse across the species while anchored to standard reference entities (small molecules, metabolites, reactions and macromolecular interactions). Thus, we support the scaling of pathway views for the community of plant researchers working on the genome and transcriptome-enabled plant species. At present, Plant Reactome provides pathway projections for 82 plant species representing a wide spectrum of plant families and photoautotrophs (Figure 1, Supplementary Table S1).

The Plant Reactome extensively collaborates with various plant genomics projects. The basic platform and data model was adopted and remodeled in collaboration with the Reactome (1,2). The integration of reference gene sequences and expression data sets (1) is performed collaboratively with the EMBL-EBI projects, Ensembl Plants (4) and Expression Atlas (5). Also, we utilize annotations provided by the Gene Ontology (GO), UniProt and ChEBI projects (6,7) and link to literature references in PubMed. We exchange data, best curation practices, and data-formatting protocols with other public resources including Planteome (8), MaizeGDB (9), TAIR (10) AraPort (11), Phytozome (12), Genome Database for Rosaceae (13), TreeGenes (14), Legume Information System (15), SolGenomics (16) and PeanutBase (17). Wherever possible, we link to other public resources for more in-depth information on various entities represented in our database. Plant Reactome provides access to pathway data in various standardized formats and via Application Programming Interfaces (APIs), and it encourages other public platforms to embed our pathway widget for data integration, pathway visualization and cross-referencing.

In the following sections, we summarize updates to the Plant Reactome since our last publication (2) covering Gramene release #52 (November 2016) to the current Gramene release #61 (April 2019). This summary includes new website design and knowledgebase configuration, additional pathway biocuration and projection, new features and functionalities, outreach and community training, and continued integration with other public resources (i.e. PubChem and EMBL-EBI Expression Atlas).

## PLANT REACTOME WEB SITE UPDATE

The Plant Reactome web site (https://plantreactome.gramene.org) is the primary entry point to Gramene's pathway portal. It has an entirely new, fully-responsive front-end design and includes the content revision as well as full implementation of Secure Sockets Layer (SSL). Our new homepage (Supplementary Figure S1) provides a quick search feature and links to the pathway browser, data analysis tools and download options, user guide, video tutorials, release summary, news and documentation, our publications, training materials, and APIs. We have also improved our interface for web services with Swagger (https://swagger.io). Users can navigate this website to search or browse pathways using a hierarchical schema, access and download data in standardized formats, visualize curated baseline expression of pathway-associated genes fetched from EMBL-EBI Expression Atlas (5), compare projected pathways from their favorite species with reference rice pathways, and analyze high-throughput gene expression data, as described previously (2). Plant Reactome pathway data has also been re-indexed and made available via an integrated search interface at http://gramene.org, which allows scientists to find genes through auto-suggested filters, and visualize search results via interactive views, both in aggregated form and in the context of a gene. A Powered-by-CyVerse (18) mirror of the Plant Reactome knowledgebase is also available at https://plantreactome.cyverse.org.

## BIOCURATION OF REFERENCE RICE PATHWAYS

Plant Reactome's versatile and flexible data model allows the synthesis and conceptualization of pathways by utilizing heterogeneous information while acknowledging gaps in the current knowledge. Thus, besides metabolic, transport, hormone signaling, and gene regulatory pathways, the model allows the depiction of complex processes, such as plant development, organ differentiation, and responses to stress conditions. We continue to utilize manual biocuration of genes and gene products, reactions, pathways and processes, and extract protein annotations from the UniProt (Swiss-Prot/TrEMBL) database using an automated script. In addition, our curators utilize GO annotations for assigning molecular function, subcellular location, and biological process to the reference entities, add cross-references to the ChEBI (7), PubChem (19), miRBase (20) and Ensembl-Gramene databases (1), and integrate information about a gene or gene product's structure-function, subcellular location, mutant phenotype, and pathways summary based on a survey of the literature. The subcellular location of proteins is assigned based on the published studies or predictions from CropPAL (21) and TargetP (22). We submit all manually curated data to the central database maintained by the human Reactome, and for each Plant Reactome release, we extract the desired plant subset marked for public release to build a new version of the database. This strategy allows curators of both projects to utilize a large repertoire of small molecules, biochemical and other common elements without duplicating informatics and biocuration efforts.

At present, we host 1824 gene products mapped to 1723 reactions associated with 298 manually curated reference rice pathways. It reflects an increase of 76 reference pathways, 698 reactions and 651 reference gene products since our last update (Supplementary Table S2). We have integrated the majority of metabolic pathways from Pathway Tools-based (23) RiceCyc (24) into Plant Reactome with added curation and curated an additional 10% new metabolic pathways not found in RiceCyc. Furthermore, we have extended manual biocuration of complex biological processes comprising multiple pathways. Considering the lack of molecular details associated with various biological processes at the protein level and the vast availability of transcriptomic data, we have successfully used our existing data model to create gene-regulatory pathways that show relationships among DNA, RNA, Protein and miRNA entities. Likewise, manual biocuration of complex biological processes was accomplished by utilizing various reaction types (i.e. association, catalysis, transcription, translation, post-translational modifications, transport and localization) linked to other sub-pathways or hormone signaling pathways. The ‘black box’ reactions are used to represent the molecular processes where exact details are unknown, but the outcomes are known from a mutant phenotype. As new details become available in the near future, such gaps will be curated.

Plant growth and developmental pathways are grouped into vegetative organ development (i.e. leaf and root formation) and reproductive organ development (i.e., flower, seed, anther, and pollen formation). The plant developmental processes were curated by combining information related to genes, transcripts, and regulatory factors (e.g., transcription factors, miRNAs, and catalytic proteins) to provide a better understanding of the biological events occurring in different cellular compartments. For example, the ‘Reproductive meristem phase change in rice’ pathway shows interactions of miRNAs (miR396, miR393, miR156, miR159, miR172, miR529), transcription factors (SPLs, MADS-box transcription factors, SNB, GAMYB, IDS1), their target genes and associated protein complexes. Similarly, transcriptional events associated with early embryogenesis and their connections to various hormone signaling pathways are shown in Supplementary Figure S2.

**Figure 2.**
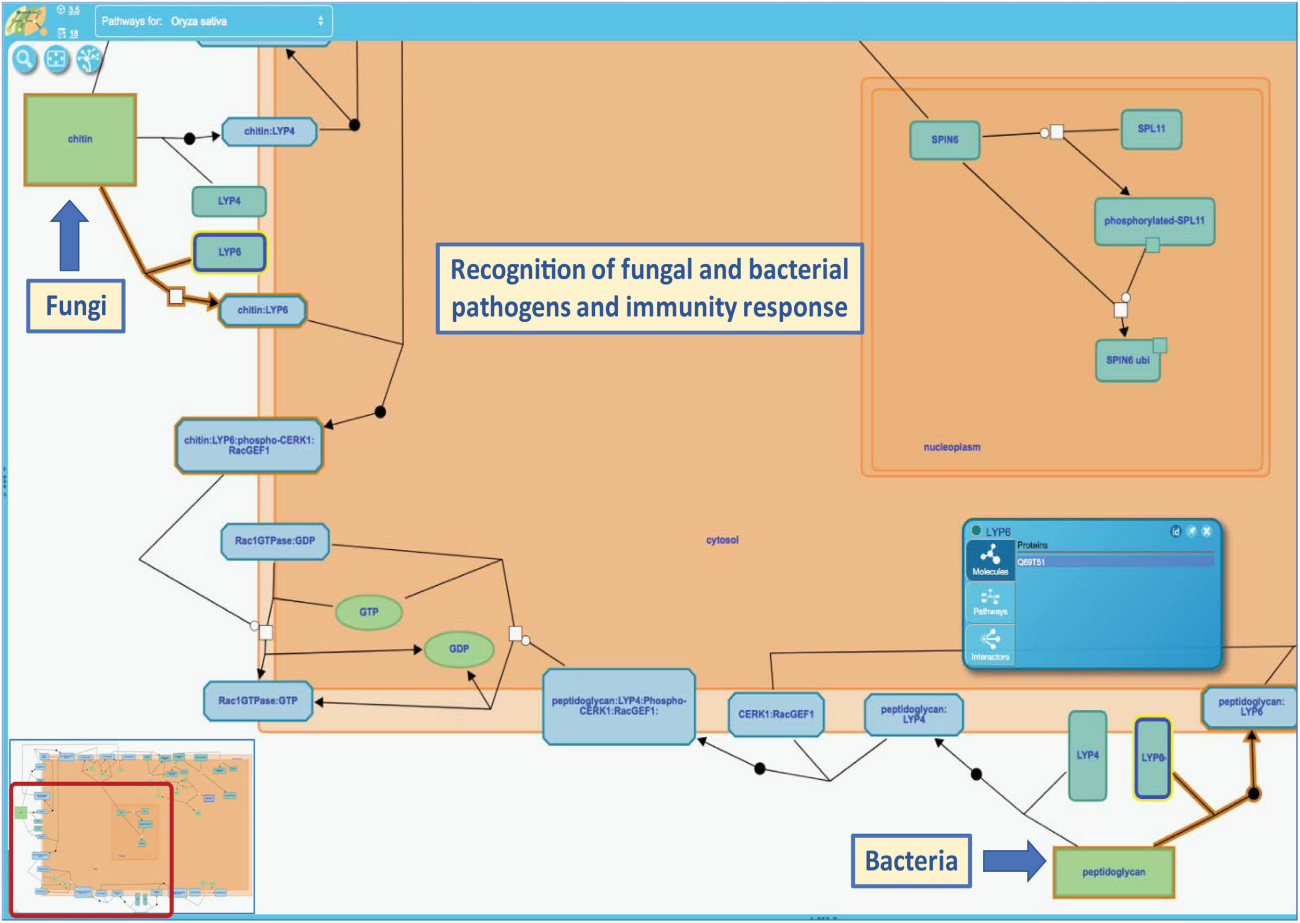
An example of a pathway depicting biotic stress response in rice. Current knowledge related to ‘Recognition of fungal and bacterial pathogens and immunity response’ has been summarized in this pathway. Depending on the type of pathogen, components of the microbial cell walls (e.g. fungal chitin, or bacterial peptidoglycan) serve as elicitors in plant innate immunity. The chitin elicitor binding protein (CEBiP) acts as the chitin receptor, whereas membrane-localized lysin motif-containing proteins 4 and 6 (LYP4 and LYP6) bind to peptidoglycan and chitin. Eventually, the binding of microbial cell wall components to plant cell membrane receptor(s) induces a downstream signaling cascade, which in turn triggers expression of regulatory factors, defense-related genes, and the components of programmed cell death leading to pathogen resistance. View pathway at https://plantreactome.gramene.org/PathwayBrowser/#/R-OSA-9611432

Pathways representing a plant's response to various stimuli or stressors are arranged under two categories: (i) response to abiotic stresses, and (ii) response to biotic stresses. The response to abiotic stress category includes pathways for the response to temperature (heat or cold), nutrient deficiency (phosphate), submergence, drought, salinity and heavy metal (aluminum, arsenic) stress. We have recently begun to curate pathways involving plant-pathogen interactions, for example, ‘Recognition of fungal and bacterial pathogens and immunity response’ (Figure 2).

## GENE ORTHOLOGY-BASED PATHWAY PROJECTIONS

We continue to utilize a combinatorial approach involving manual curation of reference rice pathways and automated gene orthology-based projection to scale up pathway knowledge to other species rapidly. Such projections provide the opportunity to expand the reach of the original curation to more plant species and allow users to upload experimental data for analysis from a broader spectrum of plant life. This list of species has now grown from 62 to 82 diverse members of the plant kingdom (Figure 1 and Supplementary Table S1) with a particular focus on global staple crops, model organisms, and species of unique evolutionary divergence. Sequence homology across species is provided by comparisons of assembled genomes or transcriptomes using two methods: (i) Compara analysis provides HMM-based sequence clustering and phylogenetic comparisons for all the genomes available in Ensembl Plants and Gramene; and (ii) Inparanoid clustering, a modified pipeline (24) developed by the Jaiswal Lab at Oregon State University, contributes ortholog data for an additional small number of species (Supplementary Table S1). The orthologs are extracted from the source databases and transformed into a series of ‘orthopair’ files. During the projection process, orthologs from other species are mapped to the reference rice reactions. If at least one ortholog is present for any reaction, that reaction, along with its parent pathway, is included in the projection. The reactions associated with a confirmed ortholog within a pathway can be represented graphically, whereas reactions lacking orthologs can be omitted pending future annotation and/or improved genome/transcriptome sequence and assembly. If more than one ortholog is retrieved for a single reference gene product, defined sets of those orthologs are created.

To assess the scope and the quality of comparative pathway projections, we used the Plant Reactome API to grab all terminal pathways from the event hierarchy for all available species and then used two Python scripts, ‘orthology_data_grabber.py’ and ‘orthology_rebuilder.py’ (available at https://github.com/PlantReactome/external-data-analysis/tree/master/orthology_stats), to generate heatmap clustering, as shown in Figure 1. In general, we find fewer orthologs in lower plants and bacteria and higher counts of orthologs for polyploids and species closely related to rice, followed by other monocot plants. However, the density of projected pathways, reactions, and gene products in any given species can be impacted by multiple factors including evolutionary distance, quality and version of the genome/transcriptome assembly and gene annotation, and ploidy of the projected species. Nonetheless, in addition to quality control checks, such comparisons can provide an evolutionary perspective for pathway analysis.

## PATHWAY BROWSER AND ANALYSIS TOOLS

Users can access the pathway browser from the homepage or from a query result. The pathway browser consists of three tightly coordinated data visualization panels (Supplementary Figure S1): (i) the left-hand panel lists pathways using a hierarchical schema, (ii) the upper right-hand panel shows the pathway diagram, and (iii) the lower right-hand panel consists of various tabs showing additional information such as, a pathway summary with literature citations, external links providing relevant information on structure and function of various biochemical entities and baseline expression of genes. Users can choose a species for display from a pull-down list and navigate a hierarchical schema of super-pathways and sub-pathways up to reactions, and explore the connections between pathways.

We continue to display baseline expression profiles of genes associated with pathway events and corresponding anatomogram images programmatically fetched from EMBL-EBI Expression Atlas (https://www.ebi.ac.uk/gxa) (5). From the expression view, users can click on a hyperlink that connects directly to an EMBL-EBI Expression Atlas page hosting available differential expression data for the genes associated with the selected reaction or pathway. Since our previous report (2), transcriptome data from 10 new species were added to the EMBL-EBI Expression Atlas, and the number of experiments analyzed for differential gene expression increased from 671 to 804.

The built-in analysis tools are accessible from the Plant Reactome homepage by clicking on the ‘Analyze Data’ icon or from the icon in the header of the Pathway Browser. As described earlier (2), users can select ‘Species Comparison’ tool to compare the reference pathways from rice with any other species of their choice that is available in the Plant Reactome. The result of pathway comparison analysis can be downloaded as a table or as images of comparative views of the pathway diagrams and in turn, can be used to identify potentially missing enzymes and/or functional reactions in orthologous species. The curators can utilize this information to revise and improve gene or pathway annotations, or researchers can investigate experimentally if these events are actually lacking in a given species due to breeding history, evolutionary process, or natural selection and adaptation.

Another analysis tool, ‘Analyze your data’ (Supplementary Figure S1), allows the uploading and analysis of user-defined large-scale expression data (transcriptome, proteome, metabolome) in the context of pathways. Formatting data requirements, step-by-step procedures for data upload, and visualization of the results, as well as the results output, were described in detail in a previous publication (2). In the following sections, we describe two new functionalities on adding the gene-gene interaction data overlay feature and the graph database implementation.

### Expansion of pathways using gene–gene interaction data overlays

Acknowledging the slow progress of manual curation of pathways, and availability of limited resources for biocuration, Plant Reactome has added a new feature to support the import and overlay of gene-gene interaction data, facilitating the exploration of new connections and providing potential new information on gene product activity or its regulation. As shown in Supplementary Figure S1, the interaction overlay key is located on the right-hand side of the pathway diagram window. This feature built around the PSICQUIC services (25) allows users to choose gene-gene interaction data available from the BioAnalytic Resource (BAR) (26), EMBL-EBI IntAct (https://www.ebi.ac.uk/intact) (27), or our internal Plant Interactome. Our internal Plant Interactome resource provides a collection of gene-gene interactions gathered from peer-reviewed publications and other well-known resources that may not be available via external data services, e.g. AraNet (28,29). The feature also allows users to upload interaction data of their choice directly from a comma- and/or tab-delimited local file (for example see Supplementary Table S3) in one of three different formats: PSI-MITAB, basic tuple format (BTF), and extended tuple format (ETF). A BTF-formatted file is a two-column data file with one interaction per row and the two interactors listed in column 1 and 2 of the same row, whereas ETF offers more options in addition to those in the BTF file, by including interactor alias, respective interactor species, score and experimental evidence. Alternatively, users may use PSICQUIC services (25) to access interaction data by providing a URL. Typically, if the gene–gene interaction data is associated with gene products listed in the Plant Reactome pathway diagram, the interactor count shows up next to the gene product box, which when expanded displays the spoke-and-wheel overlay of interactors.

**Figure 3.**
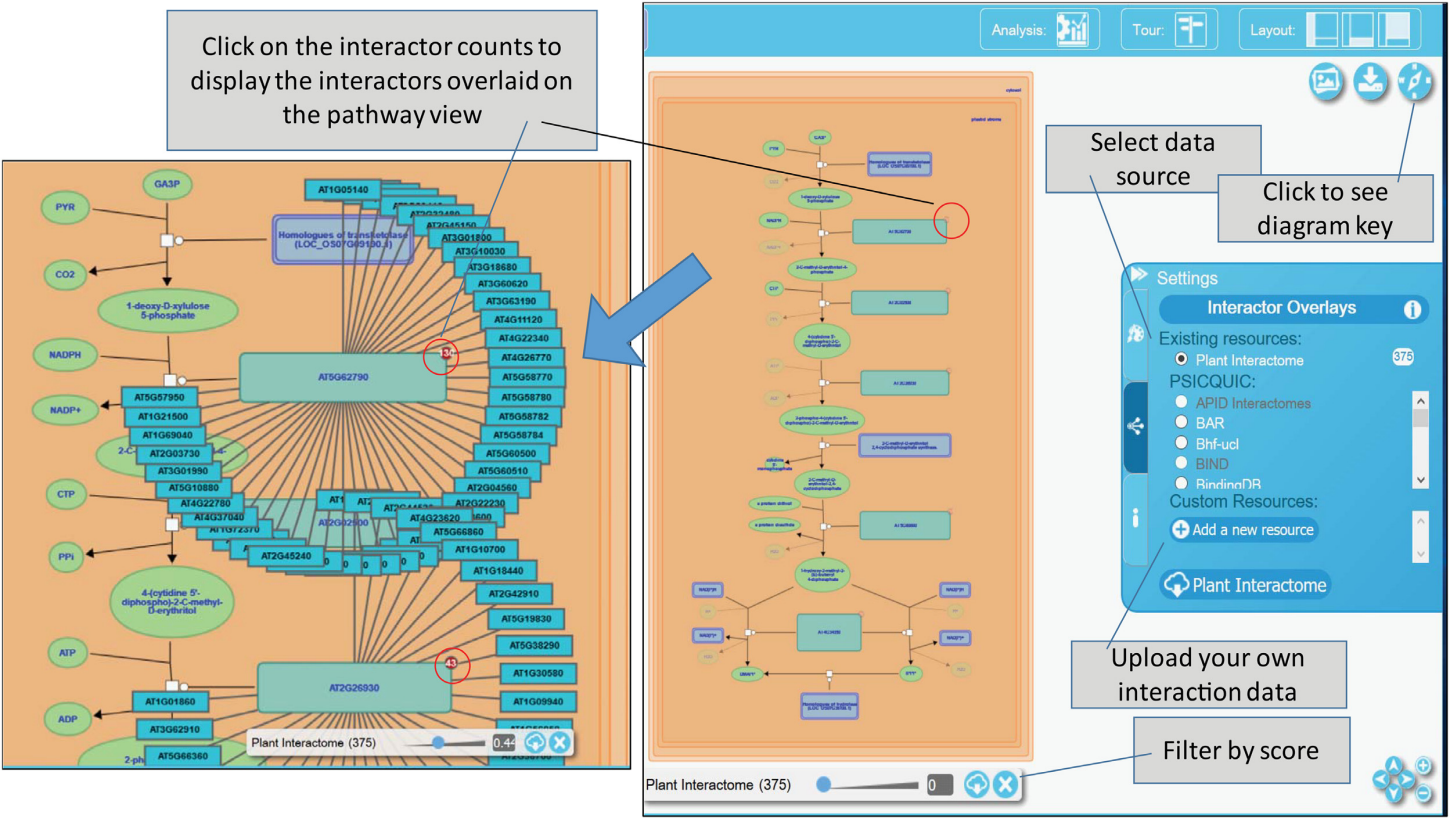
Gene-gene interaction overlay analysis for *Arabidopsis thaliana* genes encoding enzymes of methylerythritol phosphate (MEP) pathway. In the pathway diagram, the interactors associated with two enzymes (AT5G62790 and AT2G26930) are shown as a spoke-and-wheel overlay. A user has the option to display interactors of all enzymes of this pathway (total 375 from Plant Interactome and 6 from BAR) and download as a table (see Supplementary Table S3). View pathway at https://plantreactome.gramene.org/PathwayBrowser/#/R-ATH-1119464

Figure 3 shows the functionality of the interactome overlay feature on the plastid localized methylerythritol phosphate (MEP) pathway involved in the synthesis of isoprenoid precursors for carotenoids, chlorophyll and other polyprenoids. In this example, we investigated Arabidopsis gene interaction data and found 375 unique interactors from Plant Interactome and six interactors from the BAR (Supplementary Table S3). These interactors mapped to the 5 genes coding for the enzymes of the MEP pathway: AT5G62790 coding for 1-deoxy-d-xylulose 5-phosphate reductoisomerase (DXR) has 130 interactors; AT2G02500 encoding 2-C-methyl-D-erythritol 4-phosphate cytidylyltransferase (MCT) has 67 interactors; AT2G26930 encoding 4-(cytidine 5′-diphospho)-2-C-methyl-d-erythritol kinase (CMK) has 43 interactors; AT5G60600 encoding 4-hydroxy-3-methylbut-2-en-1-yl diphosphate synthase (HDS) has 134 interactors; and AT4G34350 encoding 4-hydroxy-3-methylbut-2-enyl diphosphate reductase (HDR) has 68 interactors (Supplementary Table S3 and Figure 3). Further analysis of these interactors suggests that these 5 genes interact with each other and 2 MEP pathway genes. AT2G26930 and AT4G34350 interact with 4 genes AT1G76490, AT2G38700, AT5G27450, AT3G54250 from the Mevalonate pathway (MVA). Interestingly, the HDR catalyzes a rate-limiting step of MEP pathway, interacts with hydroxy methylglutaryl CoA reductase (HMGR, AT1G76490) that catalyzes a rate-limiting reaction of the MVA pathway. Another notable interaction identified by this analysis relates to the interaction of CMK with mevalonate diphosphate decarboxylase (AT2G38700 and AT3G54250), and mevalonate kinase (AT5G27450) involved in the MVA pathway. This analysis suggests an interaction between the MVA and MEP pathways so that genes in these pathways complement or co-regulated to maintain the supply of isoprenyl precursors for compartmentalized downstream pathways. Crosstalk of the MEP pathway with cytosolic mevalonate (MVA) pathway is well studied at the level of metabolites and gene expression (30). Specifically, gene coexpression network studies support connections between MVA and MEP pathway genes (31,32). Our molecular interaction overlay feature was able to capture these connections, which are currently lacking in the curated Plant Reactome pathways, thus prompting our biocurators to review the data and update the pathway interactions in future data releases. However, users do not have to wait until the next release for additional curation, they can easily find these connections using the interactome overlays. The 5 genes of the MEP pathway also interact with a large number of additional genes involved in the biosynthesis of polyisoprenoid, tryptophan, cysteine, methionine, glutamine, UDP-glucuronate and Flavin compounds. However, further research will be needed to learn about the exact nature of interactions involved, such as protein–protein interactions, feedback interactions, and co-regulation by a common agent.

We also found that the MEP pathway genes interact with plastid localized proteases: CMK interacts with AT5G64580; DXR interacts with AT1G05140, AT4G18370, and AT2G32480; HDS interacts with AT3G18490 and AT4G25370; and HDR interacts with AT1G19740, AT2G32480, and AT1G05140. Furthermore, our interactome overlay analysis captures the interactions of MCT with Clp protease subunits ClpC1 (AT5G50920), ClpC2 (AT3G48870) and ClpD (AT5G51070), and two plant-specific accessory proteins ClpT1 (AT4G12060) and ClpT2 (AT4G25370). The Clp protease complex is composed of a catalytically active proteolytic core formed by ClpP1, ClpP3-P6 and inactive ClpR1-R4 subunits, and is stabilized by two other subunits ClpT1-T2 (33). The ClpC1, ClpC2 and ClpD subunits serve as unfolding chaperons, and ClpS1 forms an adapter complex with ClpF subunit (33–35). ClpC-mediated proteolytic degradation of deoxyxylulose 5-phosphate synthase (DXS), the first enzyme of the MEP pathway, was shown by Pulido *et al.* (36). Previous studies suggest regulation of the MEP pathway at the post-transcriptional, translational or post-translational levels (37,38), including the degradation of MEP pathway enzymes mediated by Clp proteases (39).

Similarly, users of the Plant Reactome can get information about the gene interactors that play an important regulatory role at the transcriptional, translational, or post-translational level using interactome overlay analysis. Furthermore, the data provided on gene-gene interaction are generated using diverse evidence consisting of a mix of heterogeneous methods, including published experiments and predictive computational methods. Thus, unlike expression analysis or metabolite concentration data, interaction data contain an associated confidence score, which the user can apply as a filter using a slider feature. Interactors will appear and disappear as the confidence threshold is crossed on the slider. Each displayed interactor provides a link to the appropriate Ensembl Gramene gene page, where available.

### Graph database implementation drives the knowledgebase

When the human Reactome and the Plant Reactome were first envisioned, a relational database model was the predominant choice for complex and rigorous biological data modeling. Thus, MySQL has long served as a solid foundation for describing, storing, and providing access to the biological models depicting metabolic and signaling pathways. Moving forward, as the size and scope of both the human Reactome and Plant Reactome knowledgebases have grown, performance and software design considerations have necessitated a re-evaluation of the most efficient and flexible data engine for modeling pathway space. Graph databases, born of network theory and experimental data retrieval systems, have emerged as a viable alternative to the classical relational database (40).

Graph theory helped to ground major advances in omics assembly algorithms (41,42), interaction networks, and semantic inference, thereby popularizing their development into full database platforms. Graph databases are efficient, schematically flexible, and scale well, due to an emphasis on relationships (edges) between data points (nodes), and a design that reduces the number of queries, traversals, and data joins required to return requested data (40). Neo4j (https://neo4j.com) an exceedingly popular graph database, was selected to drive internal data delivery on the Reactome platform (40). MySQL or its open-source implementation MariaDB (https://mariadb.org) is still retained as the versioned, reference data source and the foundation for curating with the Reactome Curator Tool used by our biocurators, but Neo4j has become the basis for most of the web-based data retrieval and services within the Plant Reactome. An example of an internal Neo4j server graph is shown in Supplementary Figure S3. Most internal application requests for the return of a list of pathway events, species, or gene products take the form of a query to the Neo4j service running on the webserver. This service is not directly accessible to the end-user, but can be accessed via the Content Service API, as described in the following section.

## ACCESSING PLANT REACTOME DATA

Users, depending on their comfort level and informatics skills, can access publicly-available Plant Reactome data via the pathway browser described earlier, the Content Service API, and single-file and bulk downloads of data and diagrams.

The Content Service (https://plantreactome.gramene.org/ContentService) is a RESTful web interface (https://www.ics.uci.edu/∼fielding/pubs/dissertation/rest_arch_style.htm) designed for both internal uses in the Plant Reactome knowledgebase and by external users and resources importing our data. The majority of data access within the Plant Reactome application arrives via responses from requests to the Content Service. Plant Reactome data can be requested remotely via this service. For functional examples of how to query the service, Plant Reactome has implemented a Swagger interface to document and give access to the underlying data calls (Supplementary Figure S4). For example, if a user would like a listing of all species represented in the Plant Reactome, they can query the ‘/species/main’ method documented in the Content Service, either by clicking the ‘Execute’ button found next to the method call on the Swagger interface, or by executing the demonstrated curl command on the command-line (curl -X GET ‘https://plantreactome.gramene.org/ContentService/data/species/main’ -H ‘accept: application/json’). All queries are transported via http/https protocol, and results returned from the Content Service consist of responses formatted as JSON (http://www.ecma-international.org/publications/files/ECMA-ST/ECMA-404.pdf). Other examples of service queries include requesting a hierarchical listing of all pathways and reactions for a given species (curl -X GET ‘https://plantreactome.gramene.org/ContentService/data/eventsHierarchy/Populus%20trichocarpa’ -H ‘accept: application/json’), or requesting all participating physical entities present in a pathway or reaction (curl -X GET ‘https://plantreactome.gramene.org/ContentService/data/participants/R-PTI-5632095/participatingPhysicalEntities’ -H ‘accept: application/json’). Projects using the Content Service to access Plant Reactome data include our partners within Gramene and at Ensembl Plants.

Plant Reactome data is packaged with updated content at the time of the new public release and is accessible via the ‘Download’ icon in the site header or downloadable bulk files (Supplementary Figure S1). Available formats include mapping files that variously associate stable identifiers with pathways, reactions, and genes. Compressed bulk files, containing all diagrams as SVG or PNG images and structured pathway data descriptions in the BioPAX3 or SBGN formats, are also available for download. Our collaborators at EMBL-EBI Expression Atlas, Ensembl, UniProt and PubChem, make use of these mapping and other data files to index Plant Reactome data for cross-reference and integrated functionality.

## DATA INTEGRATION WITH OTHER PUBLIC RESOURCES

Plant Reactome now offers an embeddable Diagram Widget, which allows other sites to display a dynamic, interactive pathway viewer within their own applications. All pathways are available for embedded viewing in remote web applications via the DiagramJs pathway widget. Ensembl Plants and Gramene have already incorporated this feature for providing pathway information and network diagram. Full documentation and instructions for invoking the javascript-enabled widget can be found on the Plant Reactome website (https://plantreactome.gramene.org/index.php?option=com_content&view=article&id=58). Gramene's integrated search feature has indexed Plant Reactome gene product listings and uses the Diagram Widget to display gene to pathway association in its gene search results. Using the same association mapping, Ensembl Plants embeds available Plant Reactome pathway diagrams on its individual gene pages (Supplementary Figure S5). We are collaborating with other plant genomic resources and databases to provide this functionality on their species-specific sites, where data is connected to pathways, reactions, gene products, or small molecules. Here we describe two examples of Plant Reactome data integration with external collaborators: PubChem (NCBI) displays pathway images and reaction/gene correlations in their molecular database, and EMBL-EBI Expression Atlas provides enrichment analysis for indexed genes, reactions, and pathways.

### Plant Reactome data at PubChem

PubChem (https://pubchem.ncbi.nlm.nih.gov) (19) is an open chemistry resource that provides comprehensive information on chemical substances (small molecules, biochemicals, nucleotides, carbohydrates, lipids, peptides and chemically-modified macromolecules) including their chemical-physical properties, biological activities and targets (i.e. proteins and genes), and extends information on patents, health, safety, and toxicity. PubChem has recently integrated data and annotation involving biological pathways.

In order to import and integrate pathway data from the Plant Reactome, PubChem developers first downloaded pathways in the BioPAX3 data description format (https://plantreactome.gramene.org/download/current/biopax3.zip) and the associated SVG format pathway diagram images (https://plantreactome.gramene.org/download/current/diagrams.svg.tgz) from the Plant Reactome. Subsequently, a BioPAX3 parser based on the raptor2 C library (http://librdf.org) was developed to identify small molecules, proteins and genes, and reactions from each pathway. For small molecules, they used ChEBI cross-references to PubChem compounds and proteins/genes were mapped to NCBI protein/gene identifiers via UniProt cross-references.

An example of the Plant Reactome ‘Thiamin biosynthesis’ pathway display from PubChem page (Supplementary Figure S6) shows links to the corresponding PubChem compound, protein, and gene using the molecular entities identifiers used in Plant Reactome. Detailed pathway information resides on the Plant Reactome website, yet all pathway entities ((bio)chemical, protein, or gene) are also summarized in the corresponding PubChem page. Plant Reactome is a unique contributor to PubChem that provides manually curated information on plant genes and pathways for the reference species. By enabling the integration of two resources and sharing the data and biocuration resources, we assist the community of plant researchers as well as PubChem users. All Plant Reactome pathways are (text) searchable in PubChem.

### Plant Reactome data use in the EMBL-EBI Expression Atlas

The EMBL-EBI Expression Atlas project (5) has partnered with Plant Reactome in providing baseline gene expression data (https://github.com/ebi-gene-expression-group/atlas-heatmap) as described previously (1,2). Recently, the Expression Atlas began indexing Plant Reactome pathway data so that it is searchable on their website (https://www.ebi.ac.uk/gxa/home), thus providing two-way connections between the two platforms. The Expression Atlas data can be searched with Plant Reactome stable identifiers for pathways and reactions, For example, if users type a pathway description and species, such as ‘circadian rhythm’ and ‘*Triticum aestivum*’, the search results will provide two tabs, (i) a list of experiments with baseline expression of genes associated with the query parameters and (ii) heatmap with differential expression data correlated to all annotations for that pathway from the Plant Reactome, UniProt and Ensembl Biomart (Supplementary Figure S7). This was accomplished by taking advantage of the mapping files provided by the Plant Reactome via bulk data download option. The EMBL-EBI Expression Atlas also uses Plant Reactome data (mappings of gene products/UniProt identifiers to pathways and mappings of Ensembl gene identifiers to pathways) in their production pipelines as part of their gene set enrichment analysis (GSEA) and to identify over-represented pathways associated with a set of differentially expressed genes for a particular study. Subsequently, additional mapping to associate UniProt accessions with Ensembl gene identifiers for the reference species was done using gene annotation files retrieved from Ensembl.

Based on the pathway-gene associations, Atlas provides GO terms, Plant Reactome pathways, and InterPro domain enrichment analyses and visualizations on their site including functionality to find co-expressed genes and the distribution of baseline expression across biological replicates.

## OUTREACH AND TRAINING

To assist Plant Reactome users and the broader community of plant genomics researchers, we offer onsite workshops and outreach booths at the annual Plant Biology meeting (organized by the American Society of Plant Biologists), Plant and Animal Genome conference, Maize Genetics conference’, etc. We routinely disseminate information on the project's developments via scientific journals, webinars, social media, and blog posts. We also produce recorded video-tutorials and conduct online live webinars using case studies from a variety of plant species to demonstrate the utility of the available resources, data, and tools for comparative genomics and pathway analysis (see https://goo.gl/qQ2Pjn).

Training plant biology researchers in biocuration and building a network of community curators is an important activity. Therefore Plant Reactome curators organized two Plant Gene and Pathway Curation Jamborees in 2017 and 2018. Based on our workshops, we set forth an opinion article on involving plant research community in biocuration of genes and pathways, engaging users and how biocuration training could enhance the graduate curriculum at universities (43).

We also contributed to data standards workshops organized by the DivSeek, AgBioData Consortium, Wheat-IS EWG, Grape-IS, JGI, and the International Conference of Biological Ontologies. We contributed to framing recommendations for sustainable genomics and genetics databases for agriculture that were recently published as a whitepaper by the AgBioData bioinformatics community (44).

Furthermore, we have started using Plant Reactome datasets and tools by embedding them in our courses on Plant Physiology and Functional Genomics and mentoring undergraduate students on small research projects. Since 2016, biocuration training was provided to undergraduate students. We organize and reach out to prospective young scientists by participating in activities such as the STEM Summer Camp on DNA Biology and Bioinformatics for high school students; Research and Extension Experiences for Undergraduates in Agriculture (Ag-REEU); Undergraduate Learning Experiences in working with Big Data in Agriculture; and the Biological Data Science Capstone course for graduate students at Oregon State University. In each case, students accessed Plant Reactome data via the Content Service, downloadable mapping files, and/or custom data extractions for hands-on exercises, analysis and software development, and customized the content according to the needs of the students and the participating program.

## DISCUSSION

As an open-access knowledgebase and resource for plant pathways, the Plant Reactome provides a foundational resource and an environment for learning and discovery that is accessible to plant researchers, educators, and the general public. This integrated resource provides a framework for scaling up functionalities from the reference species, *Oryza sativa*, to an additional 82 species. These functionalities include pathway browsing, analysis and visualization of omics and gene-gene interaction data in the context of plant pathways. In addition, the species comparison tool provides a phylogenetic framework to understand functional conservation and diversification of various reactions and pathways across a wide spectrum of photoautotrophs ranging from cyanobacteria to higher plant clades.

At present, the curation of reference pathways is ongoing, and our focus is on the curation of complex biological processes and their projection onto other species using the gene-orthology framework. The Plant Reactome is updated 2–3 times per year.

Plant Reactome and Gramene's commitment to free, open and FAIR access to data, open-source code, and software interoperability has led to an improved cyberinfrastructure for genomic and functional annotation, data visualization and analysis. Our streamlined user interfaces and back-end functions for the integrated search interface demonstrate these efficiencies. Plant Reactome has contributed to the open data initiative by making all its resources freely accessible in standard human and machine-readable formats. We are working with communities to frame recommended best practices to support annotations of genes and pathways, and standard workflows for functional annotation (43–45). We continue to train community biocurators, senior researchers, graduate, undergraduate, and educate K-12 students.

We have already established new collaborations with PubChem to display pathway images in correlation with their molecular database, and with the EMBL-EBI Expression Atlas to integrate Plant Reactome data in their production pipelines for gene set enrichment analysis (GSEA). We also look forward to extending collaborations with the Plant Metabolic Network (46), MapMan (47), KEGG (48) and other plant pathways, gene and small molecule data providers on sharing data and biocuration efforts. We continue to leverage the resources made available in the Powered-by-CyVerse virtual server environment by providing the Plant Reactome database mirror (https://plantreactome.cyverse.org) to facilitate training, education and integration with the CyVerse platform and user community (18).

In summary, our scientific, cyber-infrastructure, and community-building efforts and contributions have had a profound impact on the plant research community. We continue to seek feedback from users and consult the experts in reviewing curated pathways. We also invite the community to expand and enhance the model for exploring gene interactions by contributing gene-gene interaction data across the scope of the plant species we currently support.

## DATA AVAILABILITY

Plant Reactome knowledgebase webiste (https://plantreactome.gramene.org).

Plant Reactome Mirros site Powered-By-CyVerse (https://plantreactome.cyverse.org).

Training and Tutorial videos (https://goo.gl/qQ2Pjn).

## Supplementary Material

gkz996_Supplemental_File
