# Supplementary material for: Plant Reactome: a knowledgebase and resource for comparative pathway analysis
Source: Nucleic Acids Res. 2019 Nov 4;48(D1):D1093–103. doi: 10.1093/nar/gkz996 (PMC7145600; doi:10.1093/nar/gkz996)
Supplement: gkz996_Supplemental_File [file gkz996_supplemental_file.pdf]

SUPPLEMENTARY MATERIAL

Supplementary Table S1: A list of 83 plant species available in the Plant Reactome for comparative pathway analysis. The reference pathways were curated manually in rice (*Oryza sativa)* and subsequently, projected to other 82 species based on gene-orthology. The red-colored cells depict higher counts whereas shades of yellow to blue represent lower counts of the pathways, reactions, and genes/gene products. Details of an external source of data for each species (including a link) and the method employed for determining orthology are also listed. The * denotes that in the given species transcriptome assembly (instead of genome assembly) was used for pathway projection.

Supplementary Table S2: A list of updates in Plant Reactome knowledgebase covering Gramene release #52-61.

Supplementary Table S3: A list of interactors retrieved from Plant Interactome datasets for genes involved in the methylerythritol pathway (MEP). A few interactors discussed in the text are highlighted in yellow color.

Supplementary Figure S1: A view of the Plant Reactome homepage and associated functionalities.

Supplementary Figure S2: An example of a plant development pathway 'Regulation of embryo development' shows major transcriptional events associated with this developmental stage and connections to other metabolic and signaling pathways and gene products located in various organelles.

Supplementary Figure S3: Examples of internal Neo4j knowledge graph views, queries, and data formats, which are then consumed by the Plant Reactome application.

Supplementary Figure S4: Content Service Swagger interface components (https://plantreactome.gramene.org/ContentService/), consisting of a listing of available RESTful web service method calls, the query test form, the curl command that is executed to retrieve the data, and returned data in JSON format.

Supplementary Figure S5: Embeddable DiagramJs pathway widget, with constituent examples at Gramene and Ensembl.

Supplementary Figure S6: Integration of Plant Reactome data on NCBI PubChem site. An example of the thiamine biosynthesis pathway showing links to PubChem Compound, Protein, and Gene pages using the molecular entities identified in a Plant Reactome.

Supplementary Figure S7: Integration of Plant Reactome data with the EMBL-EBI Expression Atlas. Users can now search for plant pathways and associated data at the Expression Atlas website as well as access basal expression profiles of genes associated with Plant Reactome reactions and pathways. Expression Atlas also provides a "gene information card" view for individual genes and links to various Plant Reactome pathways.

Supplementary Table S1: A list of 83 plant species available in the Plant Reactome for comparative pathway analysis. The reference pathways were curated manually in rice (Oryza sativa) and subsequently, projected to another 82 species based on gene-orthology.
The red-colored cells depict a higher count whereas shades of yellow to blue represent lower counts of the pathways, reactions, and genes/gene products. Details of external source of data for each species (including link) and method employed for determining orthology are also listed. The * denotes that in the given species transcriptome assembly (instead of genome assembly) was used for pathway projection.

Taxa					Pathways	Reactions	Gene products	Sequence source	Homology method
Angiosperm	Dicot	Core Rosid	Malvid	*Arabidopsis halleri*	235	582	1021	Ensembl Gramene	Compara
				Arabidopsis lyrata	235	584	1053	Ensembl Gramene	Compara
				Arabidopsis thaliana	236	590	1059	Ensembl Gramene	Compara
				Brassica oleracea	236	581	1619	Ensembl Gramene	Compara
				Brassica napus	238	590	3209	Ensembl Gramene	Compara
				Brassica rapa	235	578	1598	Ensembl Gramene	Compara
				Citrus sinensis	240	594	2366	Phytozome	Inparanoid
				Corchorus capsularis	233	554	781	Ensembl Gramene	Compara
				Gossypium raimondii	238	602	1420	Ensembl Gramene	Compara
				Populus trichocarpa	236	596	1329	Ensembl Gramene	Compara
				Theobroma cacao	238	598	877	Ensembl Gramene	Compara
			Fabid	*Arachis duranensis*	245	624	1057	PeanutBase	Inparanoid
				Arachis ipaensis	240	594	949	PeanutBase	Inparanoid
				Cajanus cajan	240	602	1235	LegumeInfo	Inparanoid
				Cicer arietinum	241	595	1461	NCBI	Inparanoid
				Cucumis sativus	237	590	834	Ensembl Gramene	Compara
				Fragaria vesca	240	566	1014	Phytozome	Inparanoid
				Glycine max	236	599	1947	Ensembl Gramene	Compara
				Trifolium pratense	237	588	1006	Ensembl Gramene	Compara
				Jatropha curcas	213	493	534	KDRI (Kazusa)	Inparanoid
				Lupinus angustifolius	237	597	1430	Ensembl Gramene	Compara
				Malus domestica	238	576	2009	PMID: 20802477	Inparanoid
				Manihot esculenta	236	597	1180	Ensembl Gramene	Compara
				Medicago truncatula	236	587	1182	Ensembl Gramene	Compara
				Phaseolus vulgaris	239	599	1065	Ensembl Gramene	Compara
				Prunus persica	239	606	944	Ensembl Gramene	Compara
				Vigna angularis	237	582	1003	Ensembl Gramene	Compara
				Vigna radiata	226	543	891	Ensembl Gramene	Compara
		**Myrtales**		*Eucalyptus grandis*	215	501	707	Phytozome	Inparanoid
		**Vitales**		*Vitis vinifera*	238	602	930	Ensembl Gramene	Compara
		**Caryophyllales**		*Beta vulgaris*	235	576	747	Ensembl Gramene	Compara
		**Asterid**		*Actinidia chinensis*	238	594	1410	Ensembl Gramene	Compara
				Helianthus annus	237	590	1576	Ensembl Gramene	Compara
				Capsicum annuum	242	583	1214	PMID: 24441736	Inparanoid
				Coffea canephora	240	590	1050	PMID:25190796	Inparanoid
				Daucus carota	235	574	1091	Ensembl Gramene	Compara
				Nicotiana attenuata	232	507	703	Ensembl Gramene	Compara
				Solanum lycopersicum	235	583	968	Ensembl Gramene	Compara
				Solanum tuberosum	229	561	967	Ensembl Gramene	Compara
	Monocot	**Dioscoreales**		*Dioscorea rotundata*	222	480	605	Ensembl Gramene	Compara
		Poales		*Aegilops tauschii*	236	627	1123	Ensembl Gramene	Compara
				Brachypodium distachyon	231	619	1031	Ensembl Gramene	Compara
				Hordeum vulgare	234	596	1043	Ensembl Gramene	Compara
				Leersia perrieri	236	618	1001	Ensembl Gramene	Compara
				Oryza australiensis *	234	545	1704	OMAP/OGE	Inparanoid
				Oryza barthii	238	642	1057	Ensembl Gramene	Compara
				Oryza brachyantha	236	625	1013	Ensembl Gramene	Compara
				Oryza glaberrima	242	636	1051	Ensembl Gramene	Compara
				Oryza glumaepatula	239	642	1066	Ensembl Gramene	Compara
				Oryza granulata *	239	568	3509	OMAP/OGE	Inparanoid
				Oryza kasalath	191	287	365	PMID: 24578372	Inparanoid
				Oryza longistaminata *	236	604	917	Ensembl Gramene	Compara
				Oryza meridionalis	233	581	914	Ensembl Gramene	Compara
				Oryza minuta *	240	588	2092	OMAP/OGE	Inparanoid
				Oryza nivara	238	652	1066	Ensembl Gramene	Compara
				Oryza officinalis *	237	579	1882	OMAP/OGE	Inparanoid
				Oryza punctata	235	628	1067	Ensembl Gramene	Compara
				Oryza rufipogon	238	641	1076	Ensembl Gramene	Compara
				Oryza sativa Indica	242	664	1157	Ensembl Gramene	Compara
				Oryza sativa (REFERENCE)	**298**	**1723**	**1824**	**UniProt**	**Curated Reference**
				Panicum hallii FIL2	235	639	1076	Ensembl Gramene	Compara
				Panicum hallii var. hallii HAL2	237	642	1109	Ensembl Gramene	Compara
				Setaria italica	237	631	1114	Ensembl Gramene	Compara
				Sorghum bicolor	237	639	1095	Ensembl Gramene	Compara
				Triticum aestivum	238	642	3574	Ensembl Gramene	Compara
				Triticum dicoccoides	238	634	2123	Ensembl Gramene	Compara
				Triticum turgidum *	238	597	2753	PMID: 23800085	Inparanoid
				Triticum urartu	229	544	864	Ensembl Gramene	Compara
				Zea mays	235	621	1380	Ensembl Gramene	Compara
		**Zingeberales**		*Musa acuminata*	227	566	1280	Ensembl Gramene	Compara
		**Arecales**		*Phoenix dactylifera*	234	557	1097	PMID: 23917264	Inparanoid
	**Amborellae**			*Amborella trichopoda*	236	573	682	Ensembl Gramene	Compara
**Gymnosperm**				*Picea abies*	236	530	1315	Congenie	Inparanoid
				Pinus taeda	225	475	1308	TreeBase	Inparanoid
**Lycopod**				*Selaginella moellendorffii*	220	522	1226	Ensembl Gramene	Compara
**Bryophyte**				*Physcomitrella patens*	214	513	1060	Ensembl Gramene	Compara
**Chlorophyte**				*Ostreococcus lucimarinus*	156	274	226	Ensembl Gramene	Compara
				Chlamydomonas reinhardtii	164	332	273	Ensembl Gramene	Compara
**Rhodophyte**				*Chondrus crispus*	140	210	183	Ensembl Gramene	Compara
				Galdieria sulphuraria	152	253	206	Ensembl Gramene	Compara
				Cyanidioschyzon merolae	142	216	169	Ensembl Gramene	Compara
**Cyanobacteria**				*Synechocystis pcc6803*	157	311	263	Jaiswal	Inparanoid

Supplementary Table S2.
A list of updates in Plant Reactome knowledgebase (Gramene release #52-61)

Gramene release #	Pathway curation and projection			Website, tools and coding update
#52 (November, 2016)	Metabolism, regulation, and transport	Auxin transport<br>-Intracellular auxin transport<br>-Circadian Rhythm<br>-Tryptophan biosynthesis	Polar auxin transport	
	Growth and Development	Inflorescence development<br>-Long day regulated expression of florigens<br>-Short day regulated expression of florigens<br>-Transition from vegetative to reproductive shoot apical meristem	-	
	Response to biotic and abiotic stimuli	Response to stress<br>-Response to cold temperature	-	
	New species	*Beta vulgaris*, *Brassica napus*, *Syncechocystis pcc6803*, and *Trifolium pratense* (total 66 species).		
#55 (September, 2017)	Metabolism and regulation	-	-	-Several new data files made available for download that articulate the relationships between gene products, pathways, and reactions, and external reference sources such as ChEBI, Ensembl, NCBI, and UniProt.<br>-Plant Reactome pathway data re-indexed and made available via Gramene search.
	Growth and Development	-	-	
	Response to biotic and abiotic stimuli	-	-	
	New species	No new species added (total species 66)		
#56B (Feb, 2018)	Metabolism and regulation	-Photorespiration<br>-Ureide biosynthesis<br>-Beta-alanine betaine biosynthesis<br>-Proline biosynthesis V from arginine<br>-Phospholipid biosynthesis I<br>-Sphingolipid metabolism<br>-Mugineic acid biosynthesis<br>-Iron uptake and transport in root vascular system	-Cardiolipin biosynthesis<br>-Ent-kaurene biosynthesis	Plant Reactome pathway data re-indexed and made available via Gramene search.

		-Proline degradation<br>-Glutamate degradation<br>-Phenylalanine degradation III<br>-Lysine degradation II<br>-Threonine degradation<br>-Response to iron deficiency		
	Growth and Development	Vegetative structure development<br>Reproductive structure development<br>-Anther and pollen Development<br>-Primary root development		
	Response to biotic and abiotic stimuli	Response to submergence<br>-Underwater shoot and internode elongation<br>-Response to phosphate deficiency		
	New species	*Cucumis sativus*, *Dioscorea rotundata*, *Helianthus annuus*, *Corchorus capsularis*, Lupinus angustifolius, *Nicotiana attenuata*, *Chondrus crispus*, and *Galdieria sulphuraria* (total 74 species).		
#59(October, 2018)	Metabolism and regulation	-Tricin biosynthesis<br>-Lycopene catabolism<br>-Oryzalyde A biosynthesis<br>-Abscisic acid homeostasis<br>-Reactive oxygen species homeostasis<br>-Generation of superoxide radicals<br>-Removal of superoxide radicals<br>-Valine degradation<br>-Cysteine degradation	-	The Plant Reactome web site redesigned on the Joomla Content Management System.<br>Interactome overlay feature improvement: users can filter gene-gene interaction data a confidence score.<br>EBI-Intact and BAR interactome data made available using the PSICQUIC webservices.<br>Some gene-gene interaction data integrated in Plant Reactome for *Arabidopsis thaliana*.<br>The latest Plant Reactome pathway data has been re-indexed and made available via Gramene search.
	Growth and Development	-Reproductive meristem phase change<br>-Flower development<br>-Floral bract development<br>-Seed development	-	

	Response to biotic and abiotic stimuli	-Recognition of fungal and bacterial pathogens and immunity response	-	
	New species	*Daucus carota, Vigna radiata, Vigna angularis*, and *Triticum dicoccoides* (total 78 species).		
#60(Feb, 2019)	Metabolism and regulation		Thiosulfate disproportionation III	Plant Reactome pathway data re-indexed and made available via Gramene search
	Growth and Development	-Regulation of embryo development<br>-Maternal tissue PCD<br>-Cell cycle regulation<br>-Aleurone layer formation<br>-Regulation of seed size<br>-Regulation of leaf development	Flower development	
	Response to biotic and abiotic stimuli	-Arsenic uptake and detoxification<br>-HSFA7/HSFA6B transcription network induced by drought and ABA<br>-SNAC1 transcription network involved in drought and salinity tolerance		
	New species	*Arabidopsis halleri* (total 79 species).		
#61<br>(April, 2019)	Metabolism and regulation	Phytic acid biosynthesis (lipid independent)	-	SSL (Secure Sockets Layer) has now been implemented on Plant Reactome.<br><br>Plant Reactome pathway data has also been re-indexed and made available via Gramene search.
	Growth and Development	Development of Root hair	-	
	Response to biotic and abiotic stimuli	-Response to aluminum stress<br>-Root-specific gene-network of -NAC10_TF induced by drought, salinity, and ABA	-SNAC1 transcription network involved in drought and salinity tolerance<br>-HSFA7-regulatory network induced by drought and ABA	
	New species	*Actinidia chinensis* (kiwi fruit), *Panicum hallii FIL2* ("Hall's panicgrass"), and *Panicum hallii var. hallii HAL2* (total 82 species).		

Supplementary Table S3:
A list of interactors retrieved from Plant Interactome datasets for genes involved in methylerythritol pathway (MEP). A few interactors discussed in the text are highlighted in yellow color.

ID A	ID B	SCORE	#EVIDENCES
AT2G26930	AT2G35390	0.63	1
AT2G26930	AT2G44530	0.63	1
AT2G26930	AT5G66860	0.63	1
AT2G26930	AT4G23620	0.63	1
AT2G26930	AT1G32380	0.63	1
AT2G26930	AT1G10700	0.62	1
AT2G26930	AT1G18440	0.62	1
AT2G26930	AT5G19830	0.62	1
AT2G26930	AT2G42910	0.62	1
AT2G26930	AT5G38290	0.62	1
AT2G26930	AT1G30580	0.6	1
AT2G26930	AT1G09940	0.59	1
AT2G26930	AT5G14470	0.59	1
AT2G26930	AT2G38700	0.59	1
AT2G26930	AT3G01640	0.59	1
AT2G26930	AT3G54250	0.59	1
AT2G26930	AT1G56050	0.59	1
AT2G26930	AT1G58290	0.58	1
AT2G26930	AT4G16130	0.58	1
AT2G26930	AT3G42850	0.58	1
AT2G26930	AT1G01220	0.58	1
AT2G26930	AT2G31250	0.58	1
AT2G26930	AT2G17265	0.58	1
AT2G26930	AT3G06580	0.58	1
AT2G26930	AT2G47420	0.58	1
AT2G26930	AT2G47020	0.58	1
AT2G26930	AT1G31910	0.58	1
AT2G26930	AT5G66360	0.57	1
AT2G26930	AT5G27450	0.57	1
AT2G26930	AT1G33330	0.57	1
AT2G26930	AT3G62910	0.55	1
AT2G26930	AT1G01860	0.54	1
AT2G26930	AT3G55400	0.4	1
AT2G26930	AT3G02060	0.37	1
AT2G26930	AT2G02500	0.34	1
AT2G26930	AT1G63680	0.34	1

AT2G26930	AT5G64150	0.33	1
AT2G26930	AT5G22800	0.32	1
AT2G26930	AT5G64580	0.31	1
AT2G26930	AT2G27760	0.28	1
AT2G26930	AT3G18880	0.25	1
AT2G26930	AT1G49400	0.24	1
AT2G26930	AT2G43030	0.24	1
AT2G02500	AT1G63970	0.63	1
AT2G02500	AT3G04820	0.61	1
AT2G02500	AT3G48330	0.58	1
AT2G02500	AT4G14930	0.58	1
AT2G02500	AT5G50240	0.58	1
AT2G02500	AT1G72880	0.56	1
AT2G02500	AT4G02120	0.54	1
AT2G02500	AT2G29560	0.53	1
AT2G02500	AT2G34890	0.52	1
AT2G02500	AT1G30820	0.51	1
AT2G02500	AT4G20320	0.48	1
AT2G02500	AT2G36530	0.48	1
AT2G02500	AT3G12670	0.47	1
AT2G02500	AT5G50340	0.45	1
AT2G02500	AT1G74030	0.45	1
AT2G02500	AT3G28860	0.44	1
AT2G02500	AT1G55140	0.44	1
AT2G02500	AT2G31170	0.44	1
AT2G02500	AT4G12060	0.44	1
AT2G02500	AT3G13740	0.44	1
AT2G02500	AT3G54690	0.44	1
AT2G02500	AT4G25370	0.43	1
AT2G02500	AT2G19870	0.43	1
AT2G02500	AT1G26920	0.4	1
AT2G02500	AT3G45450	0.4	1
AT2G02500	AT5G50920	0.39	1
AT2G02500	AT5G38830	0.38	1
AT2G02500	AT3G48870	0.37	1
AT2G02500	AT1G16340	0.36	1
AT2G02500	AT1G79500	0.36	1
AT2G02500	AT3G02110	0.36	1
AT2G02500	AT2G14890	0.36	1
AT2G02500	AT2G45280	0.36	1
AT2G02500	AT5G56760	0.35	1
AT2G02500	AT3G56300	0.35	1
AT2G02500	AT2G26930	0.34	1
AT2G02500	AT1G55920	0.34	1

AT2G02500	AT5G51070	0.33	1
AT2G02500	AT4G18740	0.33	1
AT2G02500	AT4G35640	0.33	1
AT2G02500	AT5G14920	0.33	1
AT2G02500	AT3G13110	0.33	1
AT2G02500	AT2G17640	0.33	1
AT2G02500	AT5G60600	0.33	1
AT2G02500	AT3G57040	0.33	1
AT2G02500	AT5G12250	0.32	1
AT2G02500	AT4G24510	0.3	1
AT2G02500	AT5G16000	0.3	1
AT2G02500	AT5G48900	0.28	1
AT2G02500	AT5G62790	0.28	1
AT2G02500	AT3G18880	0.28	1
AT2G02500	AT1G67430	0.28	1
AT2G02500	AT1G27400	0.28	1
AT2G02500	AT2G05790	0.27	1
AT2G02500	AT4G34350	0.26	1
AT2G02500	AT4G34730	0.26	1
AT2G02500	AT1G52190	0.25	1
AT2G02500	AT3G20230	0.24	1
AT2G02500	AT1G22740	0.23	1
AT2G02500	AT3G19820	0.23	1
AT2G02500	AT1G65010	0.23	1
AT2G02500	AT2G24020	0.23	1
AT2G02500	AT5G11420	0.23	1
AT2G02500	AT2G45850	0.21	1
AT2G02500	AT1G58440	0.21	1
AT2G02500	AT3G24800	0.21	1
AT2G02500	AT3G49260	0.21	1
AT5G62790	AT3G63190	0.63	1
AT5G62790	AT5G58780	0.63	1
AT5G62790	AT2G45150	0.63	1
AT5G62790	AT5G58782	0.63	1
AT5G62790	AT5G58784	0.63	1
AT5G62790	AT2G32480	0.63	1
AT5G62790	AT5G60500	0.63	1
AT5G62790	AT5G58770	0.63	1
AT5G62790	AT1G62430	0.63	1
AT5G62790	AT3G18680	0.63	1
AT5G62790	AT2G23400	0.63	1
AT5G62790	AT3G10030	0.63	1
AT5G62790	AT2G23410	0.63	1
AT5G62790	AT4G22340	0.63	1

AT5G62790	AT4G26770	0.63	1
AT5G62790	AT4G11120	0.63	1
AT5G62790	AT5G60510	0.63	1
AT5G62790	AT3G60620	0.63	1
AT5G62790	AT3G01800	0.63	1
AT5G62790	AT2G17570	0.63	1
AT5G62790	AT1G05140	0.63	1
AT5G62790	AT5G10160	0.62	1
AT5G62790	AT4G29060	0.62	1
AT5G62790	AT5G05520	0.62	1
AT5G62790	AT4G29540	0.62	1
AT5G62790	AT2G04560	0.62	1
AT5G62790	ATCG00160	0.62	1
AT5G62790	AT3G03600	0.62	1
AT5G62790	AT2G22230	0.62	1
AT5G62790	AT4G05210	0.61	1
AT5G62790	AT4G21220	0.61	1
AT5G62790	AT3G11070	0.6	1
AT5G62790	AT3G25740	0.6	1
AT5G62790	AT1G13270	0.6	1
AT5G62790	AT2G25100	0.6	1
AT5G62790	AT2G45240	0.6	1
AT5G62790	AT4G37040	0.59	1
AT5G62790	AT1G72370	0.59	1
AT5G62790	AT4G22780	0.57	1
AT5G62790	AT5G10880	0.54	1
AT5G62790	AT3G01990	0.53	1
AT5G62790	AT2G03730	0.51	1
AT5G62790	AT1G69040	0.46	1
AT5G62790	AT5G57950	0.45	1
AT5G62790	AT1G21500	0.45	1
AT5G62790	AT5G04740	0.42	1
AT5G62790	AT3G02730	0.42	1
AT5G62790	AT1G33040	0.41	1
AT5G62790	AT5G19940	0.4	1
AT5G62790	AT2G38040	0.4	1
AT5G62790	AT5G53580	0.39	1
AT5G62790	AT3G16250	0.38	1
AT5G62790	AT3G46740	0.38	1
AT5G62790	AT3G18390	0.38	1
AT5G62790	AT1G63970	0.38	1
AT5G62790	AT1G76825	0.37	1
AT5G62790	AT4G09650	0.37	1
AT5G62790	AT4G18370	0.37	1

AT5G62790	AT3G51800	0.36	1
AT5G62790	AT5G63420	0.36	1
AT5G62790	AT3G57150	0.36	1
AT5G62790	AT3G62120	0.36	1
AT5G62790	AT5G19620	0.36	1
AT5G62790	AT1G12420	0.35	1
AT5G62790	AT4G31850	0.35	1
AT5G62790	AT1G12800	0.35	1
AT5G62790	AT1G12900	0.34	1
AT5G62790	AT5G08340	0.33	1
AT5G62790	AT5G23330	0.33	1
AT5G62790	AT1G76720	0.32	1
AT5G62790	AT3G12290	0.32	1
AT5G62790	AT1G33810	0.32	1
AT5G62790	AT5G52520	0.32	1
AT5G62790	AT3G51510	0.32	1
AT5G62790	AT2G21385	0.32	1
AT5G62790	AT4G37510	0.31	1
AT5G62790	AT2G04400	0.31	1
AT5G62790	AT1G76810	0.31	1
AT5G62790	AT3G52750	0.31	1
AT5G62790	AT5G14260	0.31	1
AT5G62790	AT3G27850	0.3	1
AT5G62790	AT3G09210	0.3	1
AT5G62790	AT3G27830	0.3	1
AT5G62790	AT5G58330	0.3	1
AT5G62790	AT1G03630	0.3	1
AT5G62790	AT2G42220	0.29	1
AT5G62790	AT5G53860	0.29	1
AT5G62790	AT2G31350	0.29	1
AT5G62790	AT3G47460	0.29	1
AT5G62790	AT2G02500	0.28	1
AT5G62790	AT3G12080	0.28	1
AT5G62790	AT5G48220	0.28	1
AT5G62790	AT2G28605	0.28	1
AT5G62790	AT2G01350	0.28	1
AT5G62790	AT4G35250	0.28	1
AT5G62790	AT4G18270	0.28	1
AT5G62790	AT1G19150	0.28	1
AT5G62790	AT1G49530	0.27	1
AT5G62790	AT2G23800	0.27	1
AT5G62790	AT1G03310	0.27	1
AT5G62790	AT1G02150	0.27	1
AT5G62790	AT2G34630	0.27	1

AT5G62790	AT1G31180	0.26	1
AT5G62790	AT5G14200	0.26	1
AT5G62790	AT1G80560	0.26	1
AT5G62790	AT4G26860	0.25	1
AT5G62790	AT5G07020	0.25	1
AT5G62790	AT1G75200	0.25	1
AT5G62790	AT2G37270	0.25	1
AT5G62790	AT3G22425	0.25	1
AT5G62790	AT4G04640	0.25	1
AT5G62790	AT2G39590	0.25	1
AT5G62790	AT5G66120	0.25	1
AT5G62790	AT1G06130	0.25	1
AT5G62790	AT3G11940	0.24	1
AT5G62790	AT4G39120	0.24	1
AT5G62790	AT1G09830	0.24	1
AT5G62790	AT3G19490	0.24	1
AT5G62790	AT2G18710	0.24	1
AT5G62790	AT3G46040	0.24	1
AT5G62790	AT1G50200	0.24	1
AT5G62790	AT4G14910	0.24	1
AT5G62790	AT1G07770	0.23	1
AT5G62790	AT4G25080	0.23	1
AT5G62790	AT2G05830	0.23	1
AT5G62790	AT5G59850	0.23	1
AT5G62790	AT1G70760	0.22	1
AT5G62790	AT1G48420	0.22	1
AT5G62790	AT5G52970	0.21	1
AT5G62790	AT1G20020	0.2	1
AT5G60600	AT3G19630	0.62	1
AT5G60600	AT2G39670	0.6	1
AT5G60600	AT1G60230	0.6	1
AT5G60600	AT1G17410	0.58	1
AT5G60600	AT5G39960	0.56	1
AT5G60600	AT5G63310	0.54	1
AT5G60600	AT3G02760	0.52	1
AT5G60600	AT5G03406	0.5	1
AT5G60600	AT3G18680	0.49	1
AT5G60600	AT5G45160	0.49	1
AT5G60600	AT4G23900	0.47	1
AT5G60600	AT3G10030	0.47	1
AT5G60600	AT3G63190	0.46	1
AT5G60600	AT4G11010	0.46	1
AT5G60600	AT5G65720	0.44	1
AT5G60600	AT3G03600	0.41	1

AT5G60600	AT3G01800	0.41	1
AT5G60600	AT4G22340	0.4	1
AT5G60600	AT1G76825	0.39	1
AT5G60600	AT5G10880	0.39	1
AT5G60600	AT1G76720	0.37	1
AT5G60600	AT1G62430	0.37	1
AT5G60600	AT1G79470	0.36	1
AT5G60600	AT5G39620	0.36	1
AT5G60600	AT5G52520	0.35	1
AT5G60600	ATCG00160	0.35	1
AT5G60600	AT2G25210	0.34	1
AT5G60600	AT1G16350	0.34	1
AT5G60600	AT5G60510	0.34	1
AT5G60600	AT5G08340	0.34	1
AT5G60600	AT2G17570	0.34	1
AT5G60600	AT5G23330	0.34	1
AT5G60600	AT1G44910	0.33	1
AT5G60600	AT2G02500	0.33	1
AT5G60600	AT5G60500	0.33	1
AT5G60600	AT4G05050	0.33	1
AT5G60600	AT5G11560	0.33	1
AT5G60600	AT1G21160	0.33	1
AT5G60600	AT4G29060	0.32	1
AT5G60600	AT4G34350	0.32	1
AT5G60600	AT1G07140	0.32	1
AT5G60600	AT5G19940	0.32	1
AT5G60600	AT1G78620	0.31	1
AT5G60600	AT3G63490	0.31	1
AT5G60600	AT3G46100	0.31	1
AT5G60600	AT5G61410	0.31	1
AT5G60600	AT1G76810	0.31	1
AT5G60600	AT2G36880	0.31	1
AT5G60600	AT4G26770	0.31	1
AT5G60600	AT3G60620	0.31	1
AT5G60600	AT5G14460	0.31	1
AT5G60600	AT1G80620	0.3	1
AT5G60600	AT5G21020	0.3	1
AT5G60600	AT3G52580	0.29	1
AT5G60600	AT3G44620	0.29	1
AT5G60600	AT4G24190	0.29	1
AT5G60600	AT3G47220	0.29	1
AT5G60600	AT2G36160	0.28	1
AT5G60600	AT3G11510	0.28	1
AT5G60600	AT3G09680	0.28	1

AT5G60600	AT2G31810	0.28	1
AT5G60600	AT4G25370	0.27	1
AT5G60600	AT3G11940	0.27	1
AT5G60600	AT1G61580	0.27	1
AT5G60600	ATCG00650	0.27	1
AT5G60600	AT2G39590	0.27	1
AT5G60600	AT5G03850	0.27	1
AT5G60600	AT3G54210	0.27	1
AT5G60600	AT2G33450	0.26	1
AT5G60600	AT1G30230	0.26	1
AT5G60600	AT2G01110	0.26	1
AT5G60600	AT1G43170	0.26	1
AT5G60600	AT5G13630	0.26	1
AT5G60600	AT2G38540	0.26	1
AT5G60600	AT3G46040	0.26	1
AT5G60600	AT5G04590	0.26	1
AT5G60600	AT1G71500	0.26	1
AT5G60600	AT4G40040	0.26	1
AT5G60600	AT1G34360	0.26	1
AT5G60600	AT5G28750	0.25	1
AT5G60600	AT1G07770	0.25	1
AT5G60600	AT2G44050	0.25	1
AT5G60600	AT3G27850	0.25	1
AT5G60600	AT1G08830	0.25	1
AT5G60600	AT5G02960	0.25	1
AT5G60600	AT5G59850	0.25	1
AT5G60600	AT4G29430	0.25	1
AT5G60600	AT3G61620	0.25	1
AT5G60600	AT3G27830	0.25	1
AT5G60600	AT1G72550	0.25	1
AT5G60600	ATCG00170	0.25	1
AT5G60600	AT4G02890	0.25	1
AT5G60600	AT4G18730	0.24	1
AT5G60600	AT1G49380	0.24	1
AT5G60600	AT2G34250	0.24	1
AT5G60600	AT2G42740	0.24	1
AT5G60600	AT3G16140	0.24	1
AT5G60600	AT5G09510	0.24	1
AT5G60600	AT1G67430	0.24	1
AT5G60600	AT1G33120	0.24	1
AT5G60600	AT1G27400	0.24	1
AT5G60600	AT1G05180	0.24	1
AT5G60600	AT1G33140	0.24	1
AT5G60600	AT5G39850	0.24	1

AT5G60600	AT1G31817	0.24	1
AT5G60600	AT5G24400	0.24	1
AT5G60600	AT5G64150	0.24	1
AT5G60600	AT5G24490	0.24	1
AT5G60600	AT3G18490	0.24	1
AT5G60600	AT1G01290	0.24	1
AT5G60600	AT3G58700	0.24	1
AT5G60600	AT3G10920	0.24	1
AT5G60600	ATCG01040	0.23	1
AT5G60600	AT2G21580	0.23	1
AT5G60600	AT5G49930	0.23	1
AT5G60600	AT4G34450	0.23	1
AT5G60600	AT5G64400	0.23	1
AT5G60600	AT3G26900	0.23	1
AT5G60600	AT1G23290	0.23	1
AT5G60600	AT4G37670	0.23	1
AT5G60600	AT1G29070	0.23	1
AT5G60600	AT1G22630	0.22	1
AT5G60600	AT2G06520	0.22	1
AT5G60600	AT1G66200	0.22	1
AT5G60600	AT4G33865	0.22	1
AT5G60600	AT4G21860	0.22	1
AT5G60600	AT4G35450	0.21	1
AT5G60600	AT2G46820	0.21	1
AT5G60600	AT4G24770	0.21	1
AT5G60600	AT4G29350	0.21	1
AT5G60600	AT4G14880	0.21	1
AT5G60600	AT1G04270	0.2	1
AT5G60600	AT5G59613	0.2	1
AT5G60600	AT4G17390	0.2	1
AT4G34350	AT5G49030	0.61	1
AT4G34350	AT5G08340	0.59	1
AT4G34350	AT5G23330	0.59	1
AT4G34350	AT1G05140	0.43	1
AT4G34350	AT2G32480	0.41	1
AT4G34350	AT1G78620	0.4	1
AT4G34350	AT1G78970	0.38	1
AT4G34350	AT1G66960	0.36	1
AT4G34350	AT1G78950	0.36	1
AT4G34350	AT1G78955	0.36	1
AT4G34350	AT5G42600	0.33	1
AT4G34350	AT1G78500	0.33	1
AT4G34350	AT5G48010	0.33	1
AT4G34350	AT5G36150	0.33	1

AT4G34350	AT3G45130	0.33	1
AT4G34350	AT4G15340	0.32	1
AT4G34350	AT2G07050	0.32	1
AT4G34350	AT1G78960	0.32	1
AT4G34350	AT4G15370	0.32	1
AT4G34350	AT5G60600	0.32	1
AT4G34350	AT3G24590	0.3	1
AT4G34350	AT3G56490	0.29	1
AT4G34350	AT2G01110	0.28	1
AT4G34350	AT2G33450	0.27	1
AT4G34350	AT2G19720	0.27	1
AT4G34350	AT5G19930	0.27	1
AT4G34350	AT4G24190	0.27	1
AT4G34350	AT5G24490	0.27	1
AT4G34350	AT5G19620	0.27	1
AT4G34350	AT1G72370	0.27	1
AT4G34350	AT2G02500	0.26	1
AT4G34350	AT3G10060	0.26	1
AT4G34350	AT5G52520	0.26	1
AT4G34350	AT5G64150	0.26	1
AT4G34350	AT4G29430	0.26	1
AT4G34350	AT2G24020	0.26	1
AT4G34350	AT3G09680	0.25	1
AT4G34350	AT3G21580	0.25	1
AT4G34350	AT1G31160	0.25	1
AT4G34350	AT1G07770	0.25	1
AT4G34350	AT5G13420	0.25	1
AT4G34350	AT1G49380	0.25	1
AT4G34350	AT3G04770	0.25	1
AT4G34350	AT5G02120	0.25	1
AT4G34350	AT5G59850	0.25	1
AT4G34350	AT4G30620	0.25	1
AT4G34350	AT5G44520	0.25	1
AT4G34350	AT4G34730	0.25	1
AT4G34350	AT3G60370	0.25	1
AT4G34350	AT5G10880	0.24	1
AT4G34350	ATCG01090	0.24	1
AT4G34350	ATCG01060	0.24	1
AT4G34350	AT1G67430	0.24	1
AT4G34350	AT1G76490	0.24	1
AT4G34350	ATCG00660	0.24	1
AT4G34350	ATCG00140	0.24	1
AT4G34350	AT1G27400	0.24	1
AT4G34350	AT4G31050	0.23	1

AT4G34350	AT2G39590	0.23	1
AT4G34350	AT1G53120	0.23	1
AT4G34350	AT2G43030	0.23	1
AT4G34350	AT5G23120	0.23	1
AT4G34350	AT1G43170	0.23	1
AT4G34350	AT1G19740	0.23	1
AT4G34350	AT5G01970	0.22	1
AT4G34350	AT5G02580	0.22	1
AT4G34350	AT5G40890	0.22	1
AT4G34350	AT4G04640	0.21	1

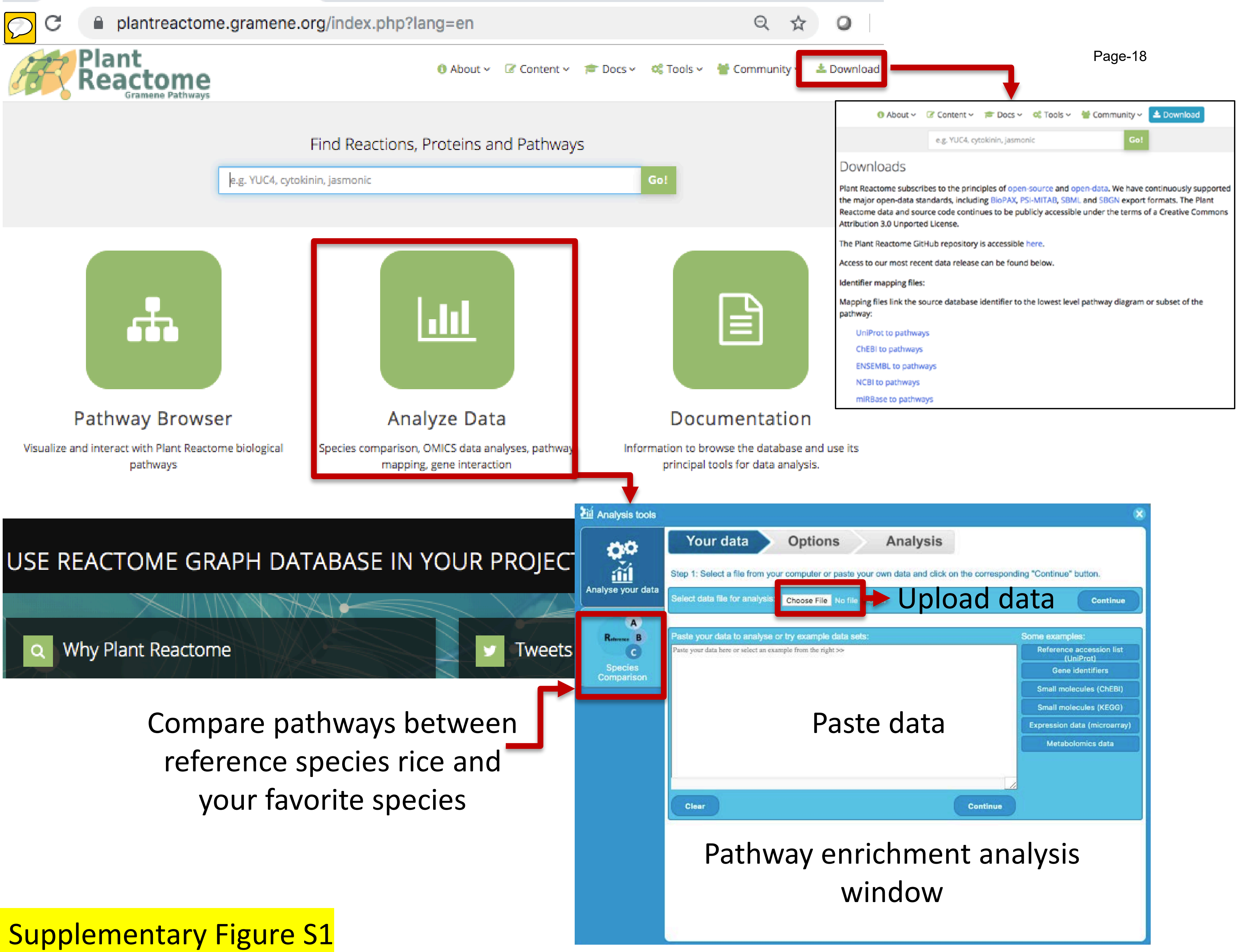

Supplementary Figure S1

Event hierarchy

Pathway diagram

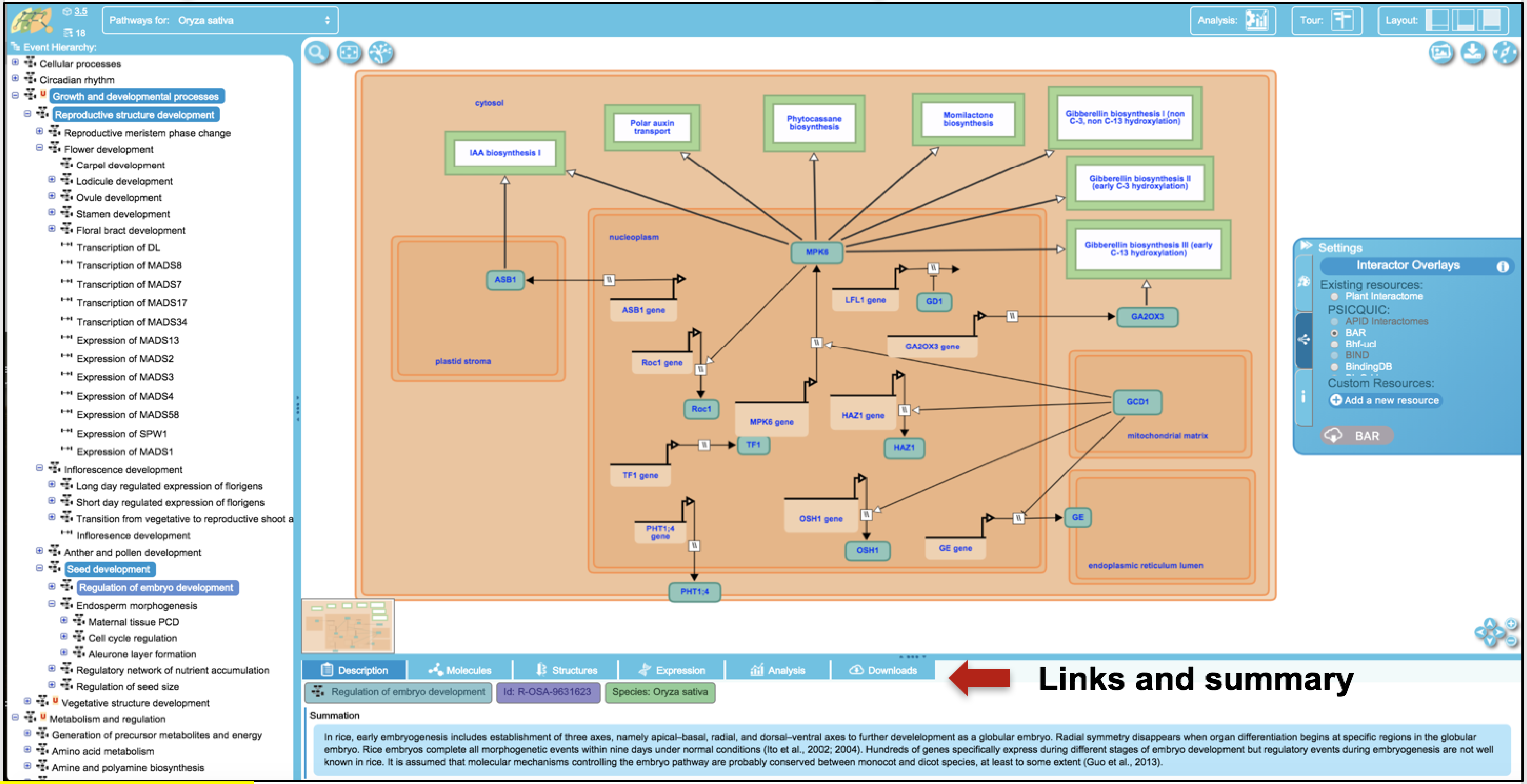

Links and summary

Supplementary Figure S2

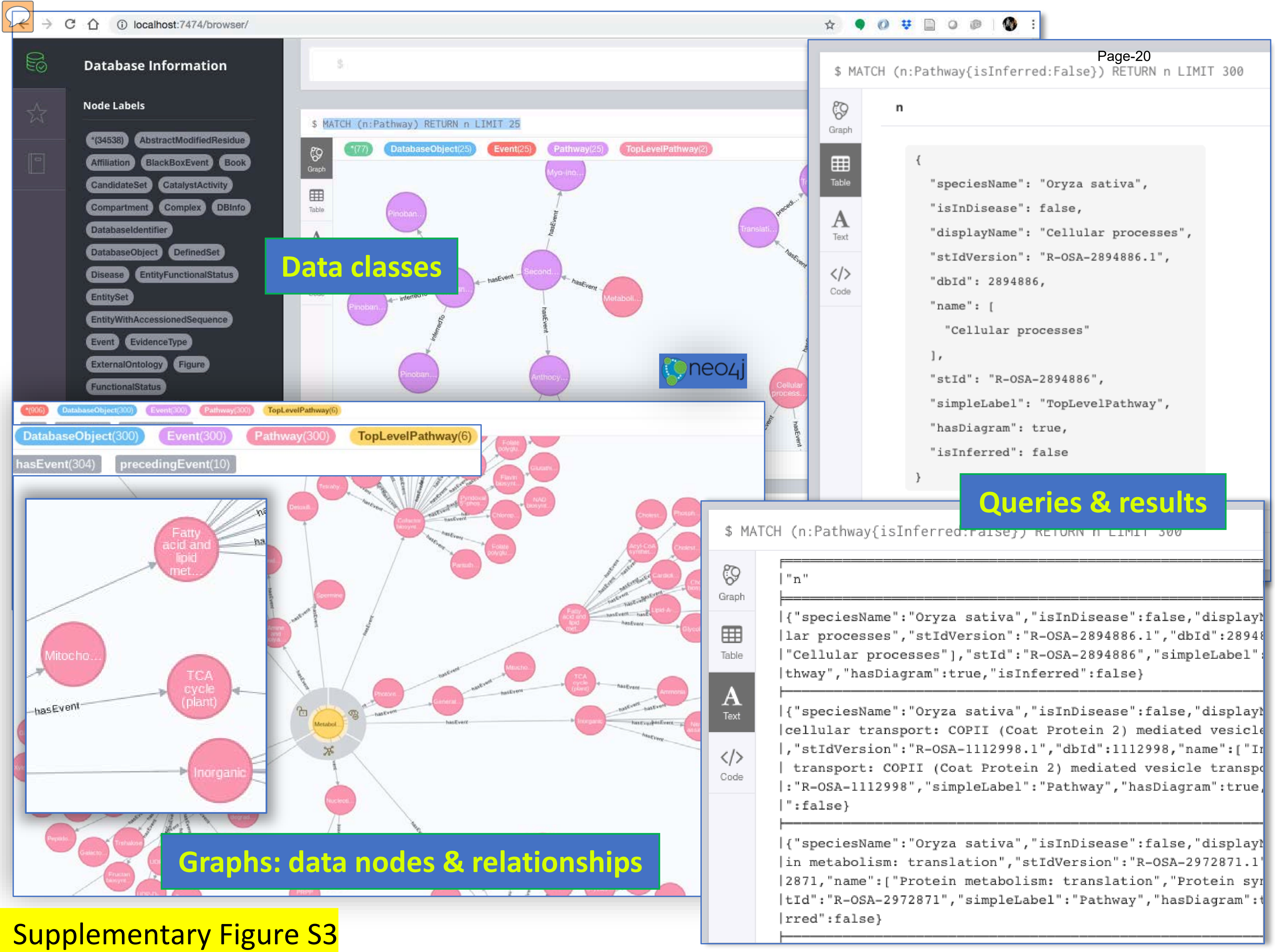

Supplementary Figure S3

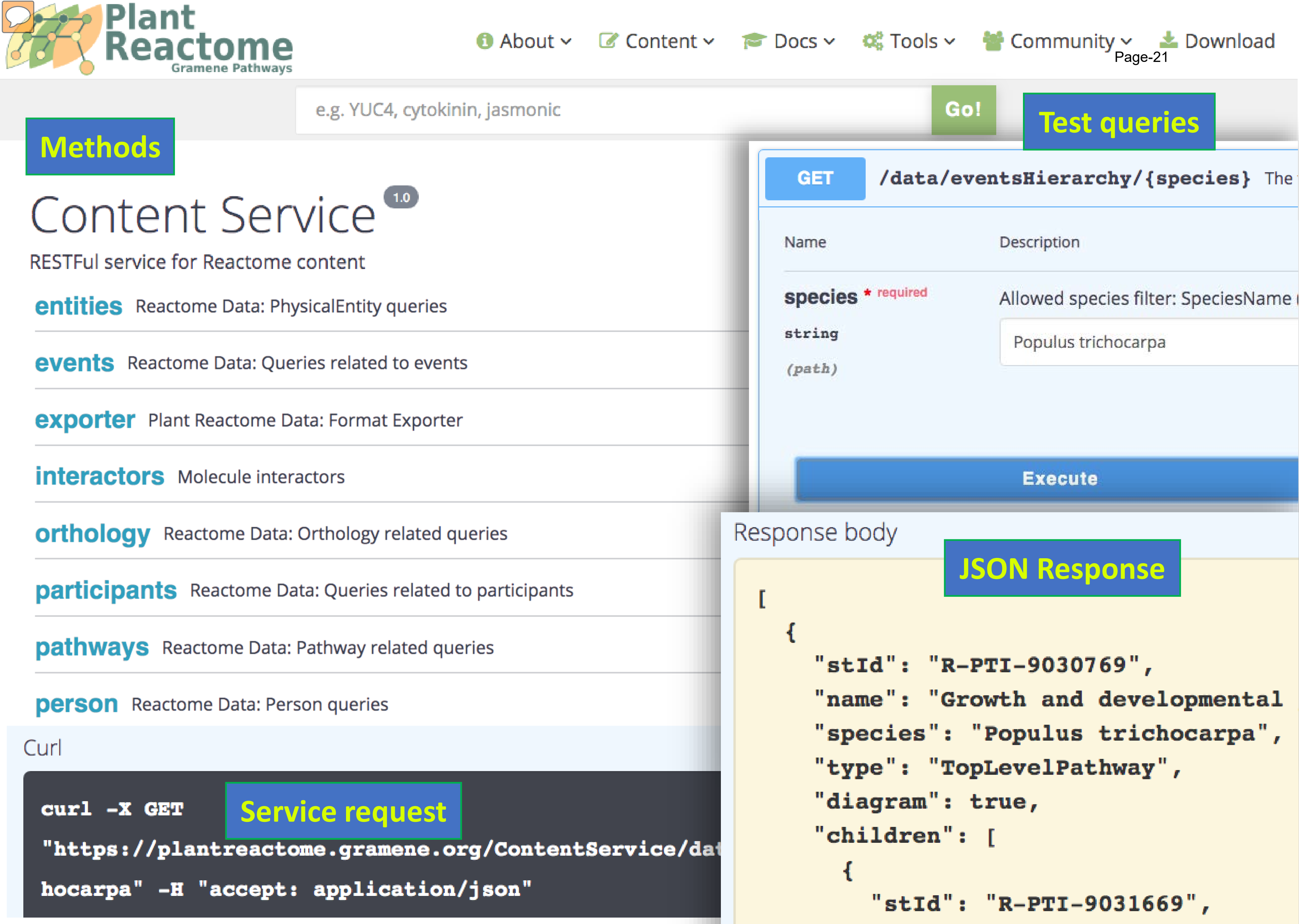

Supplementary Figure S4

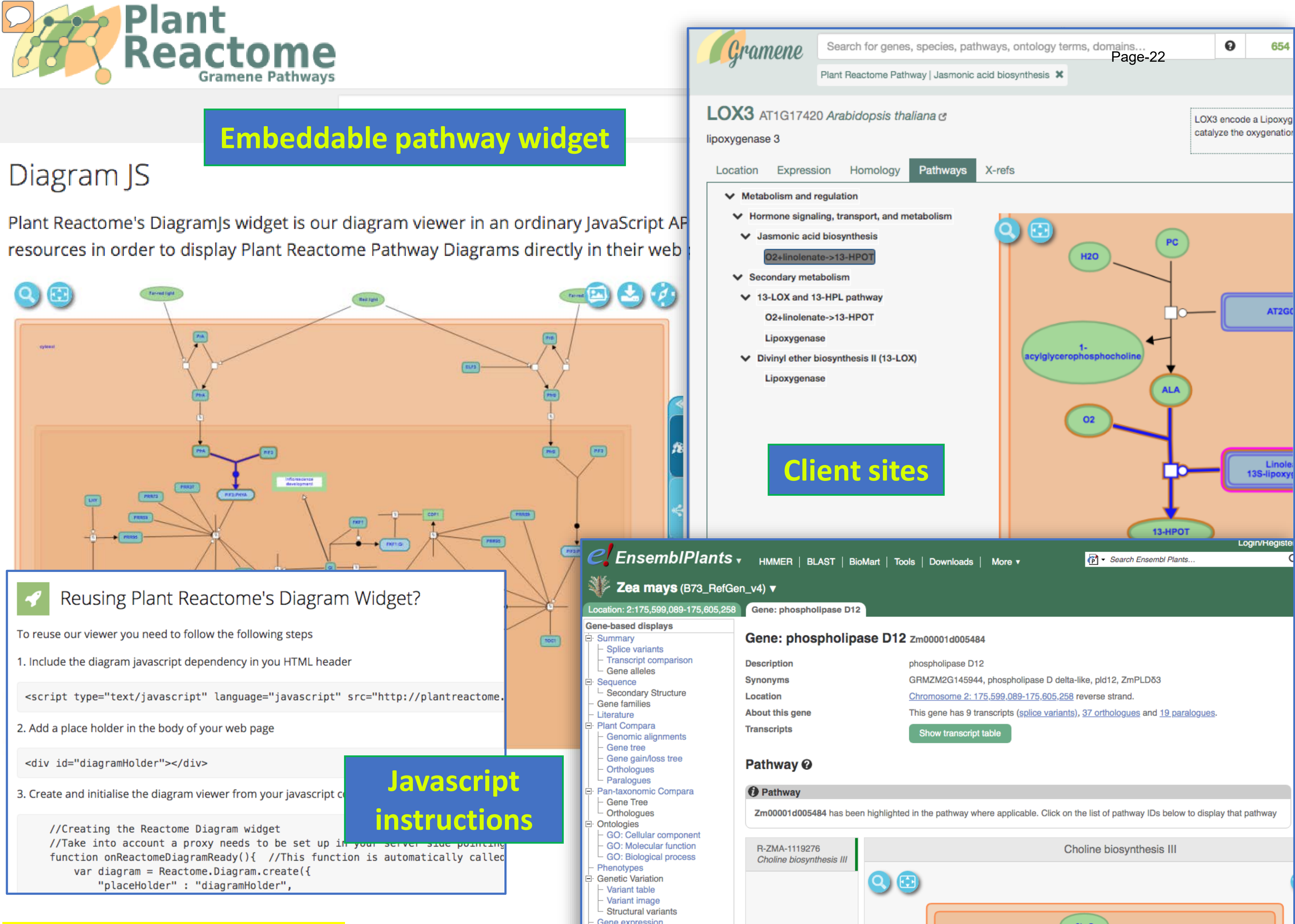

Supplementary Figure S5

Thiamin Biosynthesis
Pathway

CONTENTS

1.2 External ID

R-OSA-1119629

from Plant Reactome

2 Diagram

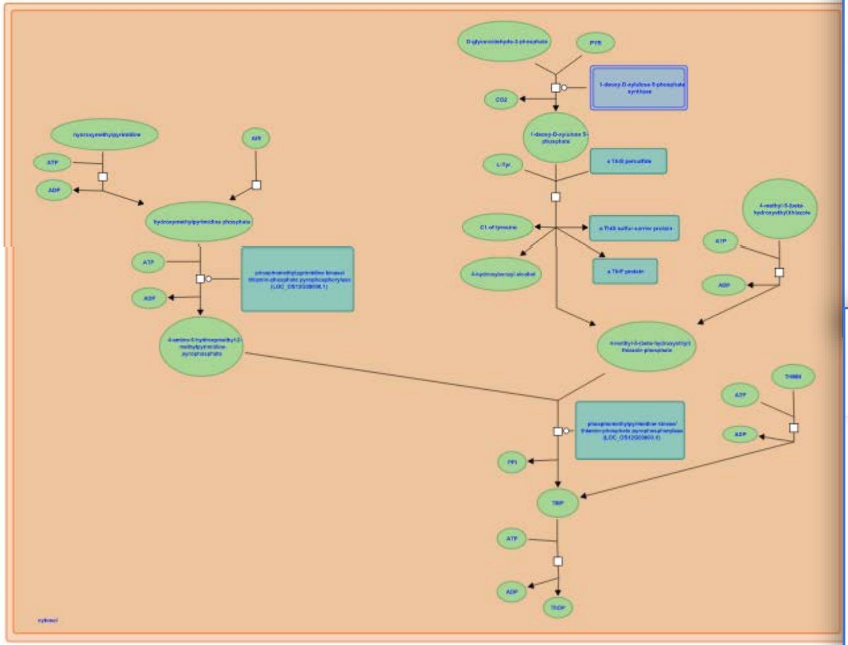

Diagram of thiamine biosynthesis

U.S. National Library of Medicine
National Center for Biotechnology Information

PubChem Probable Thiamine Biosynth... (Protein) CONTENTS

4 Pathways

3 items View More Details Download

SORT BY Pathway

Pathway	Source	External ID
Cofactor biosyntheses	Plant Reactome	R-OSA-2867929
Metabolism and regulation	Plant Reactome	R-OSA-2744345
Thiamin biosynthesis	Plant Reactome	R-OSA-1119629

PubChem LOC4351707 - Probable Thiamine Biosynthetic Bifunctional Enzyme,

3 Interactions and Pathways

3.1 Pathways

3 items View More Details Download

SORT BY Pathway

Pathway	Source	External ID
Cofactor biosyntheses	Plant Reactome	R-OSA-2867929
Metabolism and regulation	Plant Reactome	R-OSA-2744345
Thiamin biosynthesis	Plant Reactome	R-OSA-1119629

PubChem Thiami... (Compound) CONTENTS

11.2 Pathways

2 items View More Details Download

SORT BY Pathway

Pathway	Source	External ID
Thiamin biosynthesis	Plant Reactome	R-OSA-1119629
Thiamine metabolic pathways	WikiPathways	WP4297

Supplementary Figure S6

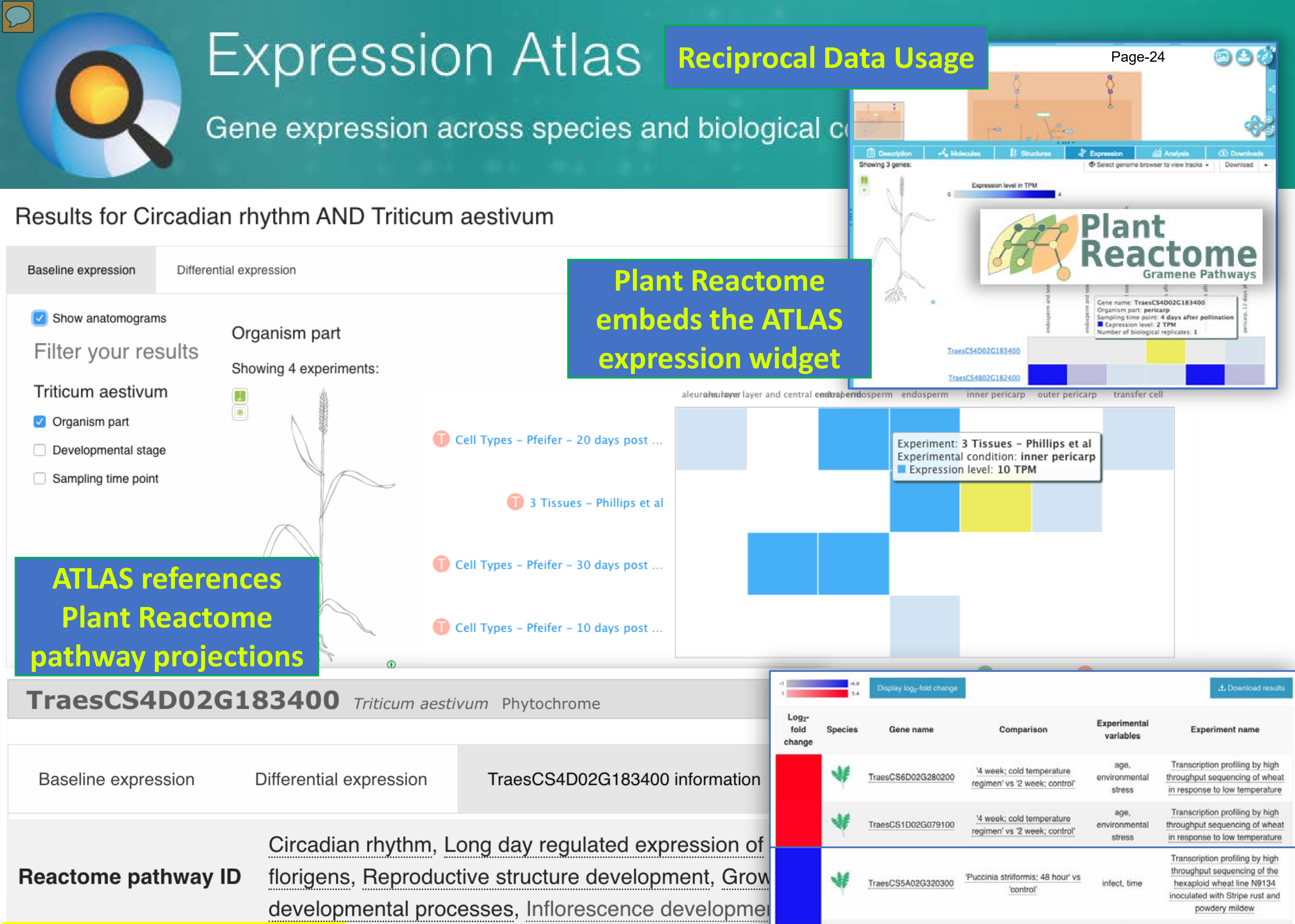

Supplementary Figure S7
